# Interactions of IDO and the Kynurenine Pathway with Cell Transduction Systems and Metabolism at the Inflammation–Cancer Interface

**DOI:** 10.3390/cancers15112895

**Published:** 2023-05-24

**Authors:** Trevor W. Stone, Richard O. Williams

**Affiliations:** The Kennedy Institute of Rheumatology, NDORMS, University of Oxford, Oxford OX3 7FY, UK; richard.williams@kennedy.ox.ac.uk

**Keywords:** kynurenine, kynurenic acid, IDO1, IDO2, indoleamine-2,3-dioxygenase, TDO

## Abstract

**Simple Summary:**

There is clear evidence that inflammation can contribute to the development of cancers, but the underlying mechanisms are not fully understood. This review focuses on metabolites of the amino acid tryptophan, especially kynurenine and related compounds, which are produced in response to inflammation and which have been implicated in cancer progression. Unfortunately, one drug that inhibits the generation of these compounds, epacadostat, has not been very successful. The review explains the actions of each of the relevant tryptophan metabolites and discusses how they interact with other compounds and biochemical pathways known to affect cancer formation. The objective is to demonstrate that the kynurenine pathway could be targeted to affect those interacting pathways indirectly and that those alternative routes could represent a means of modifying tryptophan metabolism indirectly. In either case, the range of possible targets for drugs that inhibit the link between inflammation and cancer would be expanded.

**Abstract:**

The mechanisms underlying a relationship between inflammation and cancer are unclear, but much emphasis has been placed on the role of tryptophan metabolism to kynurenine and downstream metabolites, as these make a substantial contribution to the regulation of immune tolerance and susceptibility to cancer. The proposed link is supported by the induction of tryptophan metabolism by indoleamine-2,3-dioxygenase (IDO) or tryptophan-2,3-dioxygenase (TDO), in response to injury, infection or stress. This review will summarize the kynurenine pathway and will then focus on the bi-directional interactions with other transduction pathways and cancer-related factors. The kynurenine pathway can interact with and modify activity in many other transduction systems, potentially generating an extended web of effects other than the direct effects of kynurenine and its metabolites. Conversely, the pharmacological targeting of those other systems could greatly enhance the efficacy of changes in the kynurenine pathway. Indeed, manipulating those interacting pathways could affect inflammatory status and tumor development indirectly via the kynurenine pathway, while pharmacological modulation of the kynurenine pathway could indirectly influence anti-cancer protection. While current efforts are progressing to account for the failure of selective IDO1 inhibitors to inhibit tumor growth and to devise means of circumventing the issue, it is clear that there are wider factors involving the relationship between kynurenines and cancer that merit detailed consideration as alternative drug targets.

## 1. Introduction

**Figure 1:**
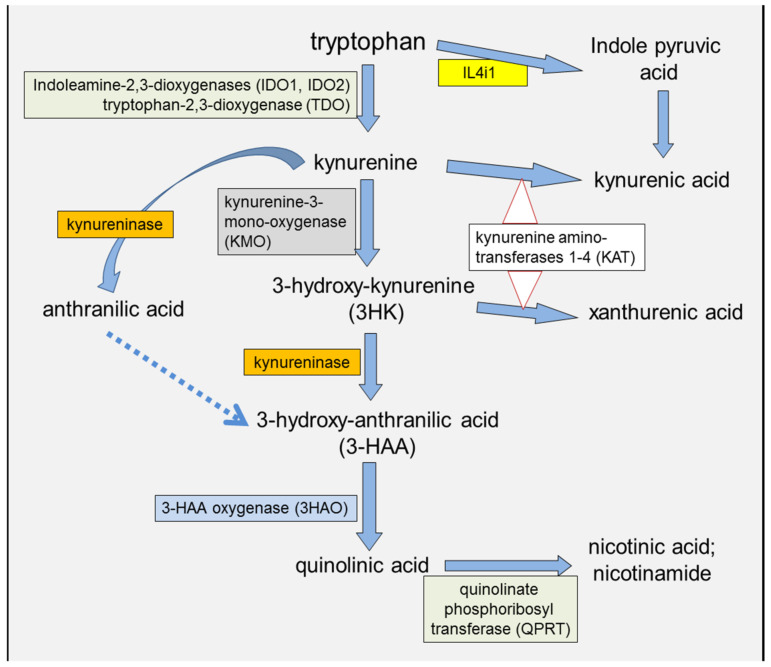
A summary of the major components of the kynurenine pathway and the enzymes responsible for their metabolism.

To help understand aspects of the relationship between inflammation and cancer, this review emphasizes tryptophan metabolism to kynurenine and downstream metabolites, a major pathway induced by immune system mediators of inflammation that includes enzymes and compounds that influence tumor initiation and growth (Figure 1). The focus will be on the bi-directional interactions with other transduction pathways and cancer-related factors to indicate the breadth of the kynurenine modulatory actions that could be affected by pharmacological treatments, especially of enzyme inhibitors. Conversely, pharmacological modulation of the kynurenine pathway could provide an indirect method of influencing other cancer-related pathways. While current efforts are proceeding to account for the failure of selective IDO1 inhibitors to inhibit tumor growth, modulating other components of the kynurenine pathway or interacting systems could provide new avenues for identifying means of disrupting the inflammation–cancer link.

## 2. IDO and Kynurenine in the Immune System

Tryptophan–kynurenine metabolism is one of the major pathways that link the immune system with the external world [1]. These environmental factors encompass not only tangible and avoidable inputs, such as tissue damage, animal and plant toxins and infectious micro-organisms, but also intangible factors such as physical or mental stress. 

**Figure 2:**
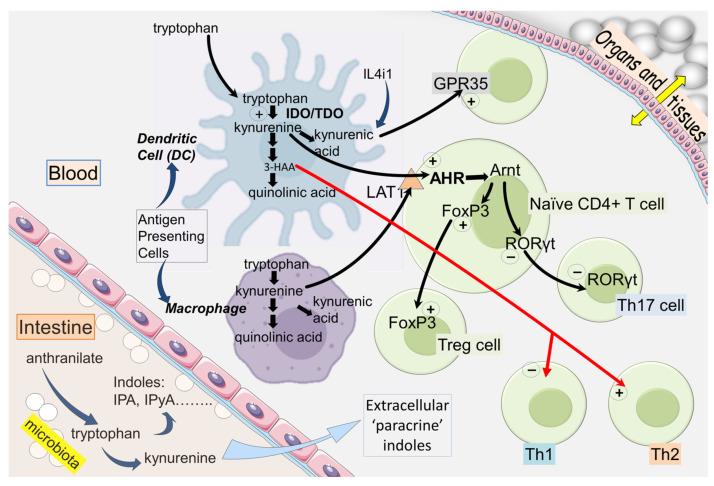
A diagram of the major cellular sites of production, uptake and actions of compounds in the kynurenine pathway and their effects in the immune system relevant to carcinogenesis. Positive signs indicate activation or enhancement, while negative signs indicate inhibition or suppression.

The first enzyme of the pathway is indoleamine-2,3-dioxygenase (IDO), two forms of which (IDO1 and IDO2) can use a wide range of indole-derived compounds as substrates and have higher affinity for tryptophan than tryptophan-2,3-dioxygenase (TDO). IDO1 is expressed constitutively primarily by antigen-presenting Cells (monocytes and dendritic cells [DCs]) [2,3,4,5], where it is induced by cytokines such as IFNγ, IL-1β or TNF [6,7]. IDO1 is also induced by molecules acting on membrane-located Toll-Like Receptors (TLRs), in particular bacterial lipopolysaccharides (LPS). The production of kynurenine by IDO (Figure 1) can then exit the APCs and function as a paracrine compound, entering other cells of the immune system, entering other peripheral cells such as endothelial cells and crossing the blood–brain barrier to affect the CNS [8]. The entry of kynurenine into cells is mediated primarily by the amino acid transporter LAT-1 (slc7a5) [4,9,10,11]. This facilitated influx, combined with kynurenine’s activation of Aryl Hydrocarbon Receptors (AHR) and the positive feedback systems discussed below, generates intracellular kynurenine concentrations up to micromolar levels, well above resting levels (Figure 2).

Free plasma tryptophan is metabolized primarily by hepatic TDO to kynurenine and downstream metabolites. With a high capacity but low affinity and a high selectivity for tryptophan as substrate, TDO is thought to be the main regulator of plasma tryptophan concentration. TDO activity is induced and increased by corticosteroids normally secreted in response to physical or mental stress. Because the kynurenine pathway has a significant impact on the CNS, including quinolinic acid as an agonist at NMDARs [12,13] and kynurenic acid as an antagonist at the same sites [8,14,15], both TDO and IDO are important links between externally modulated activity of the immune system and cognitive–psychological function. 

The protein interleukin-4-induced-1 (IL4i1) is able to oxidize tryptophan to several indole compounds such as indole-pyruvic acid, which are potent agonists at AHR [16,17,18]. They include kynurenic acid, which has been shown to be produced spontaneously from indole pyruvate by Vicenzo Politi. IL4i1 is released by tumor cells, potentially contributing to the production of host immune tolerance [19,20]. It is primarily expressed in APCs, inhibiting T cell proliferation and reducing Th1-mediated inflammation [19,21]. Being it is highly expressed in tumor-associated macrophages (TAMs), IL4i1 activity and its products reduce the activity of anti-tumor CD8+ T cells, enhancing tumor escape [18,22,23].

### 2.1. IDO and Immunological Tolerance

Lymphocyte development is associated with the deletion of cells with strong reactivity to self-antigens and the maintenance of cells responding to foreign antigens and abnormal or mutated cells. This system of self/non-self discrimination is further supported by the existence of specialized regulatory immune cell subsets, notably regulatory T (Treg) cells, which contribute to immune homeostasis by suppressing exaggerated immune responses. Normal health is dependent on the correct balance between these, as over-reactivity to self-antigens can result in autoimmune disorders, whereas underactivity can mean that subtle cellular changes and genetic mutations may escape immune system surveillance and develop into potentially cancerous states. 

The kynurenine pathway is one of the major determinants of immune tolerance. Binding of the T cell protein Cytotoxic-T-lymphocyte Antigen-4 (CTLA-4) to the B7 complex (CD80/86) on DCs induces IDO1 expression in the latter, producing anti -inflammatory molecules such as kynurenic acid and 3-hydroxy-anthranilic acid (3-HAA) [24,25,26]. Because blocking CTLA-4 (using abatacept or the antibody ipilimumab) is now a well-recognized anti-cancer treatment, it is clear that IDO1 activation is intimately involved in both autoimmune function and tumor development. 

At the sites of initial tissue damage, infection or tumor formation, the attraction of pro-inflammatory T helper cells Th1 and Th17 and Natural Killer (NK) cells establishes the earliest defense against these insults by secreting IFNγ, IL-1β, TNF and related cytokines. However, this production is inhibited by IDO, partly as a result of the local depletion of tryptophan, which activates General Control Non-derepressible-2 (GCN2), a kinase that responds to changes in the levels of loaded tRNA molecules and initiates cell apoptosis [27]. GCN2k may also activate the mechanistic Target of Rapamycin (mTOR), contributing to T cell cycle blockade and apoptosis. 

An important aspect of kynurenine biology is its relationship with T cell differentiation and polarization. Kynurenine induces expression of the transcription factor Forkhead Box P3 (FoxP3), which initiates differentiation of naïve CD4+T cells to the regulatory phenotype (Treg). In parallel, kynurenine inhibits the expression of Retinoic-acid-receptor-related Orphan Receptor-γt (RORγt), a transcription factor that promotes T cell differentiation to the more pro-inflammatory Th17 phenotype. FoxP3 is at least partly responsible for this due to preventing RORγt expression and activity [28]. The importance of this distinction is that contact between Tregs and DCs can result in several molecular interactions that regulate IDO expression, including the ligation of CTLA-4 [29]. The term ‘infectious tolerance’ describes the transmission of a tolerance-inducing state between leucocyte populations and is particularly relevant in the biology of kynurenine, as it may be passed from IDO-expressing cells to IDO-negative cells in a paracrine manner [5]. 

It should be noted that the functionally related enzymes IDO1 and IDO2 possess different structures, affinities for tryptophan and substrate selectivity. Increasing attention is being given to IDO2 as a potential alternative anti-cancer target when IDO1 inhibition is not effective [30,31,32]. It remains unclear whether the proposed non-enzymatic activity of IDO2 will compensate for inhibition of the enzyme [32]. Dual inhibitors of both enzymes are under active investigation and may prove even more suitable [31,32,33,34,35,36,37].

### 2.2. T Cell Exhaustion

The development of tumor resistance to T cell attack results partly from the exhaustion of T cell activity. Exhaustion normally develops from continuous antigen stimulation, as occurs with prolonged inflammation or cancer, or a loss of active cells [38,39,40]. There may be several mechanisms underlying the phenomenon in different situations [41]. The Activator Protein pathway (AP-1) and the level of hypoxia are involved in tumor-infiltrating T cells [42]. 

IL-15 is a highly effective activator of effector CD8+ T cells, the primary anti-tumor population of T cells, making it a valuable target to enhance anti-tumor immunity. However, tumor cells and tumor-associated macrophages express the receptor IL-15R or its α-subunit, which reduces IL-15 efficacy, giving rise to tumor resistance [38]. Macrophages in this population inhibit the expression of CX3CL1 ligand in tumor cells. Because this chemokine is an important chemoattractant for CD8+T cells, there is a fall in CD8+ tumor infiltration, which is regarded as one form of exhaustion. An IL-15R-blocking peptide therefore prevented the ligand–receptor interaction, suppressing tumor formation in a breast cancer model. The treatment also prevented tumor cell resistance to treatment with anti-PD-1 antibodies. An additional potential target arising from this study was GM-CSF (granulocyte-macrophage colony-stimulating factor), which promoted tumor cell–macrophage interaction and the induction of resistance to IL-15. Finally, in view of the role of hypoxia and hypoxia inducible factor-1α (HIF-1α) in cell function, it was noted that this protein was required for IL-15R to suppress tumor cell CX3CL1 expression.

### 2.3. Kynurenic and 3-Hydroxyanthranilic Acids

Kynurenic acid is largely anti-inflammatory, as it inhibits CD4^+^ T cell differentiation to the Th17 phenotype [43,44,45] and suppresses the release of TNF, IL-4 and IL-23 from activated monocytes [46]. It is also thought that kynurenic acid is the primary endogenous activator of G-Protein-coupled Receptor-35 (GPR35) [47,48] a protein still considered to be an orphan site, but at which kynurenate is among the most effective naturally occurring agonists. There may be additional sites of action with important roles in the regulation of mitochondrial function [49]. 

These actions will tend to promote tumor formation, but there is evidence that iono-tropic receptors exist for glutamate on leucocytes, pharmacologically similar to those in the nervous system [8,50,51,52,53,54,55] and with comparable effects on calcium movements and cell proliferation, activation, differentiation [51,56] and migration [57,58]. Glutamate induces release of anti-inflammatory IL-8, IL-10 and other cytokines from microglia and lymphocytes [50], so blockade of these receptors by kynurenic acid [51] may indirectly affect the release of several immune system mediators. The overall activity would increase inflammatory status but hinder tumor development.

3-hydroxyanthranilic acid (3-HAA) also inhibits Th1 cell proliferation and cytokine production, with a possible increase in Th2 cell activity resulting in an anti-inflammatory bias in the immune system [24,26,59,60]. No definitive ‘receptor’ has yet been identified, although the Nuclear Coactivator-7 (NCOA7) may potentially be involved [60]. Nevertheless, 3-HAA helps to stabilize CD8+ effector T cells, and it is highly redox-active. The latter property extends to an induction of the key oxidative stress regulator Heme oxygenase-1 (HO-1) [61]. Additional effects of 3-HAA are presented in the section on AHR. 

The activation of protein kinase B by 3-HAA can suppress tumor development and progression. The effect is synergistic with changes in pyruvate dehydrogenase lipoamide kinase isozyme 1 (PDK1) [62,63]. Phosphorylation of the latter enzyme in CD4+ T cells is inhibited by 3-HAA, resulting in less inhibition—effectively an increased activity—of NFκB [64]. The secondary consequences of inhibiting PDK1 include the promotion of diabetes and interference with lipid metabolism and cyclo-oxygenase activity [65]. It is therefore possible that 3-HAA could mediate a metabolic bridge between inflammation-related kinase activity, general cell metabolism and eicosanoid synthesis, all being involved in tumor stability and progression.

### 2.4. IDO Modulation and Cancer Susceptibility

Data from the Hordaland Health Study indicated the potential role of inflammation in cancer due to the consistent and highly correlated increased expression of markers such as C-reactive protein (CRP) and neopterin, with increased values for the kynurenine: tryptophan ratio reflecting increased kynurenine pathway activation [66].

An investigation of 34 plasma markers of inflammation was conducted in subjects to assess their relationship between age and inflammation, a topic clearly relevant to the increased cancer incidence, which is also age-related [67]. The analyses showed strong positive correlations between age and recognized inflammatory factors such as CRP, IL-6 and TNF, with parallel increases in IDO and kynurenine pathway activity.

Because the tolerogenic activity of IDO prevents autoimmune disease but promotes tumor formation, efforts to treat cancer have focused on the development of IDO inhibitors. However, recent work has revealed that IDO expression can be modulated by several endogenous molecules, so targeting them and affecting IDO indirectly may represent an alternative approach. Indeed, some modulators that increase IDO expression have already been linked strongly with cancer development. They include Prostate-Specific Antigen (PSA) and the cell-surface molecule CD26, which has dipeptidylpeptidase-4 (DPP-4) serine protease activity [68]. These are both included in the serine protease families with chymotryptic activity. They are also included in the group of ‘subtilase’ enzymes in view of the similarity of their substrate selectivity with that of the bacterial serine protease subtilisin. Indeed, subtilisin itself was examined partly for that reason and partly as it has already been shown to have carcinogenic potential by depleting cells of the tumor suppressors Deleted in Colorectal Cancer (DCC), neogenin and uncoordinated-5 [69,70,71]. Subtilisin proved to be as effective as mammalian PSA and CD26 [68]. 

Finally, the study included high-temperature-requirement enzyme HtrA1, one of a family of serine proteases produced by both bacterial and mammalian cells. The results showed that all four proteins increased the expression of IDO, consistent with the possibility that this could contribute to their oncogenic potential [68]. Several other serine proteases including furin, neutrophil elastase and cathepsin G did not affect IDO expression, so the phenomenon is not a feature of all serine proteases. The important implication of this work is that raised IDO activity might exist as a result of elevated levels of the active serine proteases, thus raising the susceptibility of individuals to cancer initiation. DPP-4 activity, for example, is increased chronically in subjects with diabetes, and the class of gliptin drugs are now widely used to inhibit the enzyme. The above results suggest that the incidence of cancer in those patients might be reduced as a result.

The activity of subtilisin and HtrA1 make these results especially relevant to understanding the interface between intestinal or infective microbiota and the induction of mammalian cancer. In contrast, the bacterial quorum sensor PQS (*Pseudomonas* quorum sensor; 2-heptyl-3-hydroxy-4-quinolone) inhibits the expression of both IDO1 and IDO2 [11]. It is probable that this activity may represent a significant contribution to the immunosuppression produced by pathogenic micro-organisms. Indeed, it is a topic ripe for investigation in view of the anti-cancer activity of many bacteria. A new class of drugs based on the PQS structure and concept could provide a new approach to treating cancers or reducing their development in susceptible individuals, especially in the presence of bacterial infections. 

**Figure 3:**
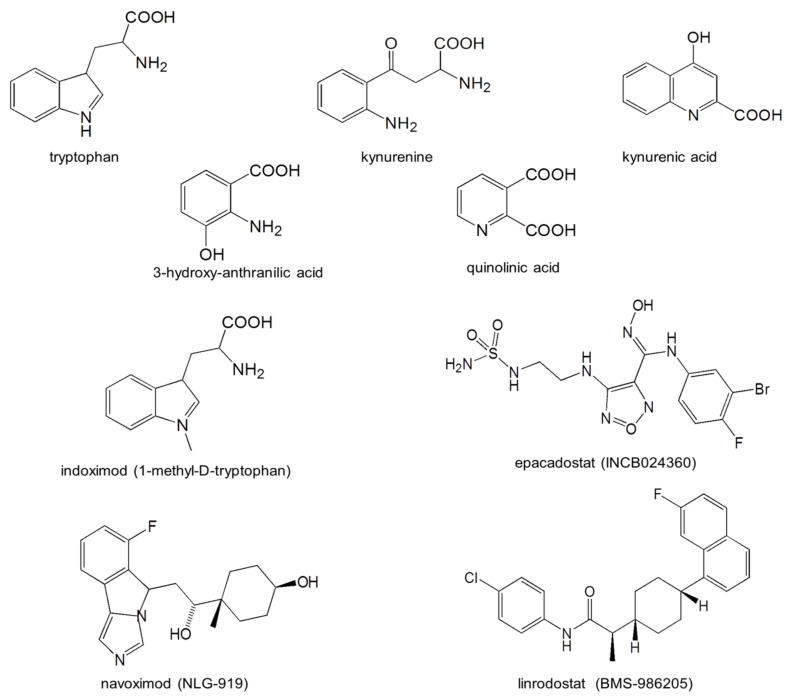
Structures of tryptophan and its major kynurenine pathway metabolites, with examples of the IDO1 inhibitors mentioned in the text.

Tumor cells are able to escape immune attack by expressing IDO or promoting its expression in the tumor environment. Efforts to inhibit immune tolerance using selective inhibitors of IDO1 (epacadostat, navoximod) (Figure 3) have been undertaken, but without much success. No doubt, this will change as newer compounds with greater potency or selectivity are produced, such as linrodostat (BMS986205) [33,34,37,72]. In addition, greater understanding of the structure of IDO, the relative activities and locations of IDO1, IDO2 and TDO, and the generation of inhibitors affecting two or three of these in parallel will likely improve therapeutic activity [73]. Alternative strategies to regulate dioxygenase activity are also under study, such as the modulation of gene alkylation. IDO1 gene expression can be inhibited by DNA methylation at the promoter, and treating mice with experimental arthritis with the DNA methyl-transferase (DNMT) inhibitor decitabine reduced the symptoms of arthritis and prevented the occurrence of relapse, in an IDO-dependent manner [74].

The discovery that IDO activity can be enhanced by the indole-derived compound N-acetyl-5-hydroxy-tryptamine, acting as a positive allosteric modulator [75], may herald a new approach to immune tolerance, complementing the inducing effects of serine proteases [68].

A different facet of kynurenine activity was noted in relation to the deterioration in efficacy of Natural Killer (NK) cells towards pancreatic tumors. This was partially prevented by 1-methyl-tryptophan (indoximod; 1-MdT) [76] and attributed to IDO inhibition, although 1-MT also inhibits the tryptophan transporter. Nevertheless, considered together with the ability of KMO to affect cell function, a potential restoration of function in the NK cell population could greatly enhance anti-cancer surveillance.

It is of course important to recognize that all tumors are not the same. As an example, anal squamous-cell carcinoma was associated with high IDO1 expression to a very high degree, correlating with a worse survival rate (88%) compared with low-IDO1-expression tumors (25%) [77]. Highly IDO1-expressing tumors such as these may be more affected by IDO1 inhibitors. More attention to this aspect of kynurenine pathway targeting in cancer might yield beneficial advances in at least some cancer conditions, perhaps leading to a more selective attack on kynurenine pathway enzymes such as KMO.

### 2.5. KMO Involvement

Although there has been a strong emphasis on understanding the role of IDO1 or IDO2 in tumor viability, there is increasing attention to downstream enzymes such as KMO. Hepatic carcinoma cells exhibit increased KMO levels, correlating inversely with survival time [78]. This was related to the enzyme’s promotion of tumor cell proliferation and migration in vitro, consistent with a role in promoting the cancer and representing a potentially valuable prognostic marker [78]. The pro-tumor activity is consistent with the increased kynurenine activating AHR and enhancing immune tolerance. The attraction of assessing KMO or other downstream enzymes such as kynureninase is that the overall profile of kynurenine metabolites generated by their inhibition would be significantly different from that produced by IDO inhibition. Notably, levels of kynurenine and kynurenic acid would be increased [79], while 3-HAA and quinolinic acid would probably be less affected, as the two branches of the kynurenine pathway via 3-HK and anthranilic acid are, to a large extent, able to compensate for the loss of one. 

Several lines of evidence have recently linked expression of quinolinate phosphoribosyl transferase (QPRT), which catabolizes quinolinic acid to nicotinic acid and nicotinamide, with cancer progression. QPRT levels are higher in many cancer patients [80]. Expression was associated with the phosphatidylinositol-3-kinase (Pi3K) and protein kinase pathway, which was suggested to mediate the negative effects, although whether the association is related to the removal of quinolinic acid or to its conversion to nicotinamide—or to both—remains unclear.

## 3. Kynurenine Pathway Interactions with Transduction Pathways

**Figure 4:**
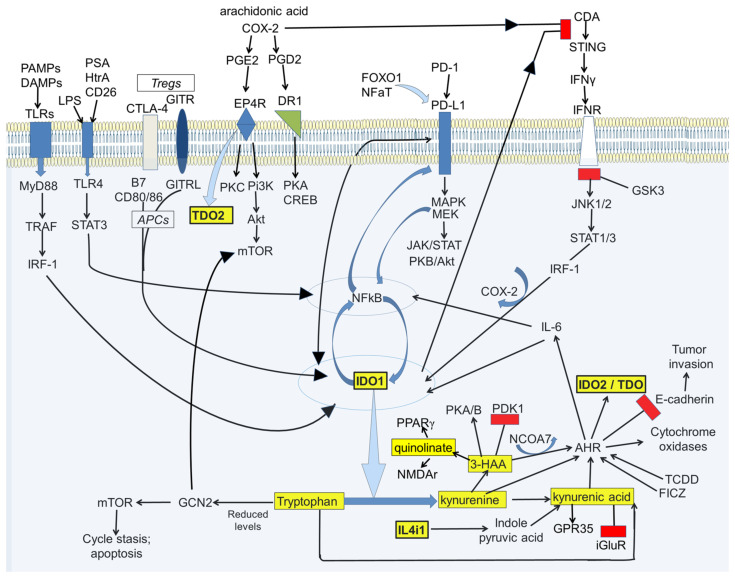
Sites of action and interactions of IDO, TDO, IL4i1 and their products of tryptophan metabolism (yellow shading) with a variety of other transduction systems cited in the text. The examples shown are not specific for any cell type, though all are relevant, directly or indirectly, to the formation or removal of tumors.

Kynurenine and its metabolites can affect several of the major recognized transduction pathways involved in cell viability and migration including AHR, the Programmed Death system (PD1-PDL1), NFκB and Fox factors (Figure 4). There are also important interactions with metabolic programs such as glycolysis and cyclo-oxygenase activity [81], as discussed in Section 4.

### 3.1. AHR

These receptors promote xenobiotic metabolism by inducing the cytochrome enzymes, and they induce IL-6, which can induce further IDO1, IDO2 or TDO in malignant cells or tumor lines [82,83,84,85,86,87,88,89]. Normally, the combination of kynurenine and AHR forms a complex with its nuclear translocator molecule (Arnt) in the nucleus to effect changes in gene transcription. There are also non-genomic mechanisms by which AHR modulates IDO expression, although these have received less attention [90]. 

The activation of AHR by kynurenine or kynurenic acid induces FoxP3, which promotes the differentiation of naive CD4+ cells to a Treg cell phenotype. AHR activation also suppresses expression of RORγt, preventing cell maturation to Th17 cells. Kynurenine and kynurenic acid are therefore at the fulcrum of an immunological see-saw that can be substantially pro-inflammatory (via Th17 cells) or anti-inflammatory (via Tregs). This balance can be modified by a wide range of other factors, which are being identified. Because the IDO–kynurenine–AHR axis produces immunosuppression via DCs and Tregs and thus limits autoimmunity, it also promotes tumor formation and progression [82,83,91,92].

The mechanisms that determine the balance between T cell types remain incompletely understood. There have been conflicting views on the effects of two of the most prominent activators of AHR, the dioxin 2,3,7,8-tetrachloro-dibenzo-dioxin (TCDD) and the endogenous photically induced tryptophan oxidation product 6-Formylindolo-[3,2-b]-carbazole (FICZ), and their roles in the generation of Treg and Th17 cells. Ehrlich et al. [93,94] explored the different kinetic behavior of these compounds, because TCDD is poorly metabolized, while FICZ is rapidly removed. At concentrations that were equivalent in terms of their activation of Cyp1A1 (a standard assay of AHR activity), both compounds induced FoxP3-negative T cells (Tr1) at day 2 of treatment and a proliferation of FoxP3+Tregs on day 10. Low levels of FICZ were still sufficient to induce Cyp1A1 but did not affect Tregs. In contrast, the same concentration of FICZ did increase the numbers of Th17 cells at an early stage of administration, whereas TCDD induced a similar response only after 10 days of administration. The data clearly indicate that the pharmacokinetic differences between the FICZ and TCDD can account fully for their apparently distinct pharmacological profiles without the need to postulate the existence of different receptors.

The AHRs also induce the expression of IL-6, a potent pro-inflammatory cytokine that not only promotes the differentiation and proliferation of Th1 cells and their production of pro-inflammatory cytokines but also induces IDO in monocytes and APCs. IL-6 is part of the important pro- and anti-inflammatory feedback circuits centered around the AHR [5,82,83,85,86]. 

In addition to its inhibition of Th1 cells, 3-HAA can also activate AHR [60], facilitating the interaction between AHR and Nuclear Co-Activator-7 (NCOA7) and enhancing the effects of kynurenine and kynurenic acid. Because the highest levels of NCOA7 are found in conventional DCs, these were cultured together with CD4+ cells. Exposure to kynurenine and 3-HAA then increased the proliferation of FoxP3+Tregs and the release of Transforming Growth Factor-β (TGF-β), both actions being dependent on NCOA7 [60]. These interactions are likely to prove significant in the development of anti-cancer agents, as NCOA7 expression is abnormal in several types of tumors and their micro- environments. An inhibitor of NCOA7 could represent a potential cancer checkpoint target. Interestingly, 3-HAA inhibits the development of tumors such as hepatic carcinoma, in vitro and in vivo, an effect involving protein kinase B and PDK1 [62,63]. As a result, 3-HAA enhances phosphatase activity and potentiates anti-cancer efficacy of sorafenib.

### 3.2. AHR and Homing

Many cytokines, particularly chemokines, induce the migration of cells to particular organs and tissues [8]. The homing of CD4+ T cells to the intestinal tract has been shown to involve AHR and their inducing expression of the chemoattractant protein GPR15 [95,96,97]. Interestingly, this activity was controlled by FoxP3 and RORγt expressed by intestinal Tregs [98]. GPR15 is also expressed in Th2 cells, where differentiation is promoted during inflammatory responses. Accordingly, GPR15 expression can be induced by the GATA3 gene in human Th2 or Treg cells [99] or inhibited by FoxP3 [100]. It is relevant that GPR15 is a prominent binding target for AHR in Treg or Th17 cells, and the combined action of FoxP3 and AHR is responsible for raising GPR15 expression in Tregs, whereas RORγt inhibits AHR binding in the Treg and Th17 subsets. FoxP3 was shown to bind to AHR and potentiate its nuclear binding at the appropriate site to induce GPR15 expression. RORγt competed with FoxP3 binding to inhibit GPR15 expression, and because intestinal Tregs exhibit higher expression of AHR compared with most other tissues, it was considered that this may relate to intestinal homing [96], although the results showed that GPR15 expression required AHR in all CD4+T helper cell populations except intestinal CD8+T. The Treg-specific ablation of AHR decreased GPR15 transcription. A positive correlation was noted between GPR15 and FoxP3 expression in human and mouse colonic Tregs, consistent with FoxP3 acting as both transcriptional repressor and activator [101,102].

The gene-binding sites for AHR also express histone modification sites H3K27Ac and H3K4me1, which may enhance GPR15 and which might link to the interaction of histone-modifying enzymes (see [103]). As noted above, FoxP3 frequently interacts with other proteins to modulate gene transcription [101,104]. However, although FoxP3 binding to DNA was not required, it cooperated with AHR to promote GPR15 expression. These results are consistent with suggestions that FoxP3 can promote transcription via AHR independently of DNA binding [104,105] to enhance GPR15. The data clearly indicate that an Ahr–FoxP3–ROR**γ**t complex influences GPR15 expression in CD4+ T cells to regulate their gut homing.

### 3.3. Programmed Cell Death Protein-1 (PD-1/PD-L1)

The receptor protein PD-1 (CD279) and its ligand PD-L1 (CD274) have attracted much attention due to the discovery of clinically useful anti-cancer antibodies or drugs such as pembrolizumab that inhibit their interaction or bind to one or the other of the two proteins [106,107,108]. The PD-L1 to PD-1 ligation affects FoxP3 stability and therefore the differentiation and function of T cells [109]. Suppressing anti-cancer CD8+ effector T cells may promote the re-activation of inactive T cells [110,111]. As for IDO, PD1 and PD-L1 are induced by IFNγ [112], and the interplay between these two systems contributes to their immunosuppressant and tumor-inhibitory activity [113]. IFNγ itself is an effective tumor inhibitor, inducing the surface expression of PD-L1, thereby contributing to cancer cell cycle arrest and dormancy. Its induction of IDO leads to the activation of AHR, which then induces stem-cell-like properties in oral squamous-cell carcinomas, promoting their dormancy [114]. The co-recruitment of IDO and PD-L1 in tumor cells is also produced by IL-27 [115], which may indicate redundancy in this activity, important to host survival, or possible synergism between them to maximize their anti-tumor potential. 

There is a broadly similar tissue distribution in IDO1 and PD-1, with parallel changes in disease stage or treatment [116,117,118,119,120] and with significant associations between them in the tumor microenvironment [121]. However, at the cellular level, their distributions appear to be distinct but overlapping, with IDO found in stromal, tumor and myoepithelial cells of patients with breast cancer, whereas PD-1 expression was localized only to the stromal tissue [112]. The expression of both genes was increased in tumors relative to normal tissue, with strong correlations between the two. Activation of PD-1 is relatively transient during infections, and at least one of its gene-promoter regulators is modified by FOXO1 as well as Nuclear Factor of Activated T cells-c1 (NFATc1), Signal Transducer and Activator of Transcription 1 (STAT1) and NFκB [104,105,106]. 

Liu Y et al. [122] highlighted that tumor-repopulating cells (TRC), a stem-cell-like group that do not proliferate but promote tumor establishment and development, drive PD-1 upregulation in CD8+ T cells through a transcellular kynurenine–AHR pathway to escape the tumor immune system. Amobi-McCloud et al. [123] have reported a direct, causal link between the PD and kynurenine systems. In ovarian cancer cells, IDO1 expression increased the number of CD8+ tumor-infiltrating T cells expressing PD-1, with a similar induction by kynurenine. The induced expression of PD-1 by kynurenine in CD8+Tc probably involved AHR, as binding sites have been demonstrated on the PD-1 gene [123]. Because the AHR is a major target of kynurenine, its involvement was examined using the antagonist CH223191, which blocked the induction of PD-1. This was consistent with kynurenine influencing the access and binding of the AHR to consensus XRE motifs in the PD-1 promoter. These results not only explain the synergistic anti-tumor activity of combinations of IDO1 and PD-1 inhibitors but also present a novel rationale for developing that concept further, either with formalized drug combinations or with bifunctional molecules able to target both systems. Although a trial combining the IDO1 inhibitor epacadostat and the PD-1 antagonist pembrolizumab showed no significant benefit [124], alternative approaches are being considered, such as using drugs with dual targets [33,34,37,72]. An improved response to the combination of IDO1 and PD-1 inhibition was anticipated by Iwasaki et al. [125], noting that expression of both PD-L1 and IDO1 were positively correlated with the presence of JAK2 and STAT1 in leiomyosarcoma. 

The effector CD8+T population exhibits a high level of nutrient uptake accompanied by increased oxidative metabolism, which underlies their proliferation and increased expression of IFNγ [126]. Activating the Glucocorticoid-Induced TNF-Receptor (GITR) overcame the effector T cell inhibition by PD-L1 in the mouse MC38 tumor model, with GITR increasing oxygen consumption. Hence, one result of activating GITR is enhancing the cell metabolism, which is required for the maintenance of CD8+T anti-tumor effector cells.

A novel suggestion arising from a study of lymph nodes was that IDO might exert distinct effects in different locations, inhibiting the immune response of tumors by depressing local effector T cell activity but altering antigen presentation by APCs and the differentiation of CD4+ T cells to relevant subsets. The results of Ishihara et al. [127] may in part reflect similar conclusions, namely that PD-L1 reflects poor survival in cases of undifferentiated sarcoma, although IDO1 expression was most closely correlated with improved survival.

Unfortunately, the promise of PD-1/IDO blockade may not benefit all cancers. Early lung adenocarcinoma tissue, for example, showed no expression of PD-L1, although it was expressed in a subgroup of patients with the highest density of tumor-infiltrating CD8+ cells, where it was located together with high IDO1 levels [128]. Only this fraction of patients may therefore be suitable for combination treatments. This variability may reflect different types or magnitudes of effect of PD-L1 and IDO1 in different tumor environments. In osteosarcoma tissue, neither protein correlated with patient outcome. The expression of PD-L1 or IDO-1 was related to the presence of CD3+ and CD4+ T cells, but only PD-L1 was present in CD8+ cells [129].

One important and highly relevant study was based on the treatment of Simian Immunodeficiency Virus (SIV) infection. It was noted that kynurenine and its metabolite levels correlate with the frequency of serious non-AIDS-related incidents, associated also with viral titers and general T cell activation [130]. The authors proceeded to treat rhesus macaques with an inhibitor of kynurenine-3-mono-oxygenase (KMO) (CHDI-340246) during the early phases of SIV infection. Confirming that the inhibitor prevented the synthesis of downstream components of the kynurenine pathway, it was noted that symptoms were improved, body weight increased and CD4+T cell numbers were raised. In addition, the expression of PD-1, which was normally present in the early response to SIV and which then correlated with disease progression, was reduced in naïve and memory CD4+ cells populations. While this result is consistent with a role for quinolinic acid in the symptoms, the levels of kynurenine and kynurenic acid were raised, emphasizing the need to consider the balance between these metabolites and their respective activity.

A metabolomics investigation of human colorectal cancer tissue revealed high levels of TDO2 and kynurenine. Either TDO or AHR was required for cells to express PD-L1 and for the expression of cancer stem cell properties. Hence, TDO and PD-L1 were usually co-expressed in the tumor cells, and the levels correlated with the presence of hepatic metastases. Looking at their differential expression and activity indicated that TDO2 was responsible for the increase in AHR levels, which then induced PD-L1 expression, again related to the production of liver metastases [131]. These changes could be prevented in PD-L1 KO mice. The data suggested that the TDO2–kynurenine–AHR interaction was responsible for the hepatic metastases of colonic cancer, probably due to inducing PD-L1 and its ability to simultaneously inhibit immune surveillance and promote cancer cell stemness.

While most of the preceding points refer to IDO1, the relative expression of IDO1 or IDO2 and that of PD-L1 or PD-L2 may have a strong influence on cancer outcome. It has been suggested that reduced overall survival was correlated with higher levels of the combinations PD-L1 and IDO2, or with PD-L2 and IDO1 [132]. Age may also be a factor, as the expression of PD-L1 and IDO2 increases with age [133]. The importance of the different enzyme forms would clearly merit closer investigation.

The close functional relationships between PD-1 and the kynurenine pathway enzymes IDO1, IDO2 and TDO are illustrated by the large number of signals that influence both checkpoints in parallel. Thus, the cytochrome enzyme CYB561D2 increases the expression of TDO2 and PD-L1, together with CCL2, suppressing T cell activity but promoting proliferation and migration, actions that were mediated via the STAT3 pathway [134].

### 3.4. NFκB

The kynurenine pathway interacts with other kinase transduction systems, but because many of those interact with each other, identifying specific links with tryptophan metabolism has proved difficult. As an example, the PD-1 system discussed above is a significant checkpoint in tumor development [135], but it interacts with several of the major kinases including MAPK, JAK, and the protein kinase B pathway involving Pi3K [81] This potential for secondary effects on the wide array of kinases and related proteins is one of the reasons why the PD-1–tryptophan connections are pivotal in cellular function. Interestingly, the promoter regions of the PD-1 gene are regulated by FOXO1 and by the Rel homology transcription factors such as NFκB and Nuclear Factor of activated T cells (NFaT) [136]. These factors, although effectively independent, do interact in the regulation of cell functions, exemplified by their roles in cardiac hypertrophy and remodeling [137]. The interface between these different systems further enhances the extent of interfering with the kynurenine pathway.

Indeed, one of the strongest links between the kynurenine pathway and inflammation is that involving NFκB, often regarded as the ‘master regulator’ and lynchpin of inflammatory activity. NFκB is particularly important in its dominant role of regulating an array of transcription factors. In one study, patients with pulmonary hypertension were found to exhibit over 800 differences in gene expression compared with control subjects, with 90 of those being relevant to regulation of the NFκB system [138]. NFκB activity tends to parallel that of IDO and related inflammatory markers such as COX-2 [139] and is now known to be a major inducer of both, acting mainly through AHR [140]. However, both IDO and kynureninase can mediate a negative regulation of the canonical NFκB system, possibly by modulating the activity of glycogen synthase kinase-3 (GSK-3), which can activate NFκB-inhibiting factors and binding proteins [141].

The link between this kinase and kynurenines was emphasized by the discovery that TDO2 in triple-negative breast cancer cells generated kynurenine, thus activating AHR. This pathway was dependent on NFκB activation [142] and was important in the cell death following detachment from a home tissue or adherent surface. The view was that the loss of cell stability induced the NFκB–TDO2–AHR axis, and this facilitated cell independence and ultimately metastasis formation in a distant site. Inhibiting TDO2 or AHR prevented this.

### 3.5. Therapeutic Implications

Delivering vesicles containing PD-L1 should activate tumor PD-1 and induce or enhance tumor cell death [138]. However, tumors also secrete PD-L1, which acts on CD8+ T cells, reducing their anti-tumor potential. The antagonist drug macitentan blocks endothelin receptors, which inhibit PD-L1 production in mammary tumors, thus preventing the demise of CD8+ T cells [143]. Combining macitentan with an anti-PD-L1 antibody improved anti-tumor efficacy by increasing the CD8+ T cell number and activity, accompanied by fewer Tregs in the tumors and draining lymph nodes of triple-negative breast cancer, colon and lung syngeneic tumor models. The anti-tumor effect of macitentan was reversed by PD-L1. The expression of endothelin receptors was strongly related to the macitentan innate anti-PD-1 resistance gene signature and the low response to PD-1/PD-L1 blockade. The results demonstrate that macitentan can improve and overcome an inadequate response to PD-1/PD-L1 blockade therapy. 

PD-1 may be relevant to the activity of trametinib, a mitogen-activated protein kinase (MEK) inhibitor used in melanoma. Trametinib has been reported to promote T cell viability and proliferation in a murine model of acute myeloid leukemia (AML) [144]. The increased T cell proliferation was associated with raised PD-L1 expression in the CD8+CD44+ population, while CD8+CD62L+ cell activation was inhibited.

Generally, tumor cell expression of PD-L1 helps the cells to avoid T cell attack while maintaining proliferation and migration. Despite the clinical success of PD-1 blockade in cancer treatment, however, response rates are low, relapse is common, and adverse effects are frequent. A novel mechanism may help to surmount these difficulties. In tumor-repopulating cells (TRC), CD8+ T cells induced the production of kynurenine consistent with the high level of IDO1 expression [122]. As it is generated, the kynurenine re-enters CD8+ cells, creating a positive feedback within which the expanding number and activation of AHRs upregulate PD-1 expression. A selective inhibitor of IDO1 in TRCs or of the AHR in CD8+ cells could be a novel route to suppress PD-1 expression.

A different therapeutic approach is to increase the efficacy of anti-PD-L1 agents [145]. Phosphorylated components of the endosomal transport systems inhibit tumor infiltration by CD8+ T cells by promoting tumor secretion of PD-L1-containing exosomes. Consequently, it was confirmed that inhibitors of phosphorylation prevented the inhibition of CD8+ cell infiltration, resulting in a reduction in mouse melanoma progression.

Cancer cell resistance to PD-1/PD-L1 inhibition continues to be a significant therapeutic problem. A screen of several varieties of cancer cells revealed compounds that prevented IFNγ induction of the IDO and PD-L1 checkpoint molecules [146]. Most of these proved to inhibit heat shock protein-90 (HSP90) and involved an enhanced stability of STAT1. Testing the HSP90 inhibitors in combination with IDO1 inhibition or anti-PD-1 agents indicated positive effects in several pancreatic cancer models.

A number of naturally occurring compounds are known to affect the expression of IDO and PD-1 or PD-L1. The flavonoid myricetin inhibited the JAK–STAT–IRF1 pathway to prevent the induction of IDO and PD-L1 by IFNγ, thereby inhibiting carcinogenesis. Myricetin also reversed the loss of Jurkat cell proliferation and IL-2 production induced by IFNγ-treated cancer cells [113]. Another natural product, erianin, inhibited PD-L1 expression and induced the lysosomal degradation of PD-L1. The mechanism involved suppression of the interaction between RAS and HIF-1α. The overall result was to invigorate cytotoxic T cells and their attack on tumor cells, with inhibition of tumor cell proliferation and migration [147]. Britannin is a compound that disrupts the interaction between Myc and HIF-1α, reducing the expression of PD-L1 and depressing the proliferation rate of tumor cells.

T cell exhaustion, discussed above, is dependent on the expression of PD-1 and PD-L1 [148]. A causal link is suggested by the rise in CD8+ activity when PD-1 is blocked, which is the basis of current anti-PD-1 therapies. 

### 3.6. Epigenetics and PD-1

With the recognition of epigenetic factors in the cause and treatment of many cancers, histone acetylation and de-acetylation have become significant pharmacological targets, with several inhibitors under study. Although often required in combination with other agents, ACY241 has been found effective against multiple myeloma [149,150]. In addition to reducing PD-1 and PD-L1 activity, this compound reduced the numbers of CD138+ cells, CD4+CD25+FoxP3+ Tregs and myeloid-derived suppressor cells (HLA-DR) Low/CD11b(+)CD33(+). Its importance for T cells lies in the ability of ACY241 to increase expression of the B7 complex (CD80/86) and MHC complexes in DCs. The compound also induced T cell co-stimulatory molecules (CD28; CD40L; OX40; CD38), generating overall a marked anti-tumor activity with enhanced IFNγ, IL-2 and TNF expression. The T cells were antigen-specific memory T cells, accounting overall for the enhancement in immune responsiveness.

### 3.7. FoxP3

Interfering directly with Tregs is one approach to inhibit tumor growth. Zammarchi et al. [151] employed an anti-CD25+ T cell antibody in a syngeneic solid tumor model, showing clear suppression of tumor growth, which was enhanced by anti-PD-1 treatment. Although there was a reduced number of Tregs in the general circulation, this was sufficiently transient to not induce autoimmune complications. A Phase I trial is underway (NCT03621982). Because CD4+CD25+ Treg cells are among the most prominent anti-tumor and pro-transplant cells, the regulation of their driving force transcription factor (FoxP3) is a potential site of therapeutic attack [152]. As FoxP3 is induced by kynurenine, it represents an important rationale for manipulation by interfering with the kynurenine pathway.

Over 100 gene mutations have been identified in FoxP3 [153,154], and post-translational modifications such as acetylation and methylation are eminently suitable targets to produce up- or downregulation of FoxP3 expression and the proliferation, robustness and stability of Tregs [104,155,156]. An important route to modifying FoxP3 activity is phosphorylation, given its susceptibility to kinases such as Lymphocyte-Specific Protein Tyrosine Kinase (LCK), which increases FoxP3 activity by phosphorylation at position Y342 [102,156,157].

The cyclin-dependent kinase CDK2 and PIM (Pro-viral Integration site for Moloney murine leukemia virus) enzymes 1 and 2 also inhibit FoxP3 activity [158,159,160]. The Pim1 phosphorylation site at S418 of FoxP3 interferes with S422, while Pim2 phosphorylates the N-terminus of FoxP3. Knockdown mouse models exhibit increased FoxP3 expression and activity [158], so promoting expression should inhibit Treg development. The utility of this approach may be limited by the non-selectivity of FoxP3 binding to various proteins [155] and the wide range of enzymes affected by the target enzymes for acetylation, methylation and phosphorylation. Acetyltransferases represent a related approach, as two typical examples, p300 and CBP, are required for optimal Treg differentiation and activity [27]. It may be possible to circumvent such problems by taking advantage of routes for modulating Treg proliferation and activity that do not involve FoxP3 [161]. 

There is a proof of concept that an inhibitor of FoxP3 complex formation with AML1 can enhance Treg function [162]. A modified FoxP3 molecule has been reported that can cross cell membranes, thus enhancing its ability to mimic the endogenous protein [163].

### 3.8. FOXO1

The transcription factor FOXO1 (Forkhead Box Other-1) is involved in the differentiation of CD4+ cells to the Th17 phenotype, while also inhibiting polarization to Tregs. These actions are prevented by FOXO1 inhibitors [113]. Wilms tumor 1 associating protein (WTAP) is expressed by naïve CD4+T c from patients with immune transplant rejection. The protein promotes FOXO1 expression, leading to increased generation of its target FoxP3 [164], and its overexpression reverses transplant rejection. 

FOXO1 has been proposed as an important indicator of the early changes in breast cancer [165]. It is also a key factor in the ability of CCL20 to recruit Treg cells and generate resistance to chemotherapy in colorectal cancer cells. The expression of CCL20 and induction of chemoresistance requires the activation of a pathway that includes FOXO1 and NFκB and correlates with patient survival [166]. Any modulation of this route by interference with FOXO1 may therefore modulate chemosensitivity of CRC and present a novel target for treatment. A similar argument applies to the differentiation of NK cells, as FOXO1 and FOXO3 are necessary for normal development of the NK lineage and innate lymphoid cells [167] by modifying the expression of IL-15Rβ. Disrupting FOXO1/3 may have widespread implications for immune system function.

A novel action of quinolinic acid is likely to be important in the understanding and treatment of glioblastoma, as it encompasses the activity of this compound in the central nervous system and the immune system. As a selective agonist at NMDA receptors in the CNS [12,13], it was likely that it would also activate these sites on leucocytes [50,53,55]. It has now been reported that it acts on NMDA receptors in macrophages to modify the FOXO1/Peroxisome Proliferator-Activated Receptor-γ (PPARγ) checkpoint pathway [168]. The result is an enhancement in immune tolerance, protective of the tumor survival. Consequently, inhibiting quinolinic acid production is a promising strategy for treating these tumors.

### 3.9. miRNA

As a means of regulating or normalizing the aberrant cell metabolism encountered in some disorders, microRNAs may become useful. miR-143 overexpression enhanced the differentiation of central memory CD8+ T cells, with reduced apoptosis and inflammatory cytokine secretion [169]. Inhibition of glucose uptake by GLUT1 appeared to be the primary target of miR-143, with the suppression of T cell glycolysis and their differentiation to Tregs. The same molecule was synergistic with HER2-CAR T cells in the induction of apoptosis in TE-7 esophageal cancer cells, which exhibited a decreased central memory T cell (Tcm) population together with reduced levels of miR-143. These may have been downregulated by IDO1 and kynurenine, because an siRNA for IDO1 increased the expression of miR-143 and Tcm numbers.

The expression of IDO1 by cancerous cells or cells in the immediate environment can inhibit T effector CD8+ cells, thus maintaining and promoting tumor growth. However, there are reports of miRNA activity on target sites directly relevant to the treatment of cancer. For instance, miR-153 inhibits IDO1 expression in colon cancer cells, potentiating the anti-cancer activity of CAR-T cells aimed at the Epidermal Growth Factor receptor [170]. Even the ectopic administration of miRNA mimics was able to suppress IDO1, preserving CD8+ effector activity [171]. 

## 4. Metabolic Interactions

An increasingly attractive method of regulating immune system or tumor cell development and activity is to interfere with basic cellular metabolism [171,172,173,174,175]. T cell activity is highly dependent on metabolic flexibility, and changes required to cope with the abnormalities of metabolism seen in tumor cells demand a corresponding flexibility (‘metabolic reprogramming’) in defensive immune system cells [174]. One advantage of this concept is that drugs are often available that target individual enzymic components of metabolism and can therefore be tested in novel combinations where appropriate. An excellent example is the combination of metformin (normally employed in the treatment of diabetes) and 2-deoxyglucose, which together inhibit oxidative phosphorylation and glucose metabolism. While both compounds separately were able to inhibit IFNγ production by activated human CD4+T cells, their combination generated unpredicted metabolic changes such as the inhibition of Myc and HIF-1α expression. This information could lead to therapies that capitalize on the information either directly or indirectly to modify T cell activity in autoimmune disorders [175]. 

### 4.1. Anaerobic Glycolysis

Anaerobic glycolysis is an energetically unfavorable process that mediates the conversion of glucose to ATP. It is much less efficient than oxidative phosphorylation but yields a variety of small-molecular-weight compounds such as amino acids and lipids required for normal cell biochemistry, many of which have significant influence on immune system cells [176]. In a study of breast cancer cells, tumor infiltration by anti-tumor NKT cells and the expression of PD-L1, IDO1 and FoxP3 were associated with high levels of glycolysis [177], an observation partly attributed to the intermediate expression of IL-17. A similar requirement for enhanced glucose and nutrient uptake, associated with increased mitochondrial activity, is typical of most T cell subtypes [178]. 

In resting cells, ATP is normally produced via the mitochondrial TCA pathway. The pathway oxidizes glucose to pyruvate or lactate (Warburg effect) in the relatively energetically expensive process of aerobic glycolysis. However, activated T cells may be prevented from employing this route by the loss of GAPDH [179]. The loss of ATP reduces the expression of cytokines such as IFNγ, an action paralleled by the direct GAPDH-mediated inhibition of T cell IFNγ gene expression. In contrast to lymphocytes, the energy supply of most tumor cells is obtained from glucose even under normoxic conditions and needs to function despite changes in local hypoxia and basic metabolic insults. This emphasizes the extent to which reprogramming could be exploited to distinguish pharmacologically between normal and tumor cells [180].

An increase in glycolytic activity can result from increased expression of the glucose uptake transporter (GLUT-1) induced by inflammatory cytokines [181]. The situation may therefore arise during microbial infections when elevated cytokines and T cell proliferation enhance glycolysis. The cellular advantages seem to lie in enhanced effector cell activity and the increased synthesis of nicotinamide in the pentose phosphate pathway [182,183,184]. However, there will be a balance between this route and the nicotinamide generation from quinolinic acid in the kynurenine pathway. As far as the author is aware, the possibility of interactions and positive or negative feedback regulation between these two sources of nicotinamide has not been investigated.

IFNγ enhances aerobic glycolysis along with inflammatory pathways dependent on PD-L1 and prevented by PD-L1 suppression [185]. However, there was no induction of tryptophan-metabolizing pathways or the JAK2/STAT1 system, consistent with a role for PD-L1 in the actions of IFNγ but not the activation of kynurenine production in relation to aerobic glycolysis.

### 4.2. Glutamine Metabolism

The metabolism of glutamine has a very significant influence on T cell biology, as the amino acid and its synthesizing enzyme glutaminase regulate expression of LAT-1, thus affecting the T cell availability of amino acids and related small molecules such as kynurenine, with secondary changes in T cell differentiation and inflammatory balance. 

The responsiveness of CD4+ and CD8+ T cells is modulated by phosphoglycerate mutase-1 (Pgam1), an enzyme essential for the glycolytic enhancement in TCR-mediated signaling, and mTORC1 activity, both of which are dependent on glutamine [186]. This is linked to T cell function and recognition of the key roles played by IL-17-secreting γdT cells, as glutamine is an important regulator [187]. Glutaminase activity and glutamine levels increase upon cell activation, and inhibitors help to restore normality in conditions such as psoriasis. The glutaminase inhibitor 6-diazo-5-oxo-L-norleucine (DON) and an in vivo bioavailable analogue inhibited T cell activation and the differentiation of CTL, Th1 and Th17 cells in autoimmune hepatitis. This was associated with reduced expression of LAT-1 (*slc7A5*) and activation of mTOR [188]. The results suggested that inhibiting glutamine metabolism could suppress T cell activation and the differentiation of pro-inflammatory T cells, with the conclusion that modulating glutaminase and glutamine activity could be directed toward the generation of an anti-tumor T cell balance. 

### 4.3. Miscellaneous Metabolic Factors

Activated cells display an increased requirement for basic elements of biosynthesis. This includes the anti-tumor CD8+ effector T cells, activity of which is induced by TCR stimulation and applies particularly to asparagine, which promotes CD8+T cell efficacy [189] by interacting with LCK. LCK is a protein tyrosine kinase that functions as an asparagine sensor, and its phosphorylation promotes TCR signaling. These interactions require upregulation of transporters accompanied by enhanced synthesis of asparagine, which results from TCR and mTOR activation [190] and which enables these anti-tumor cells to function optimally despite the local metabolic demands of the cancer cells.

### 4.4. COX and Oxidative Stress

It has long been recognized that there is an intimate relationship between the kynurenine pathway and eicosanoid metabolism. The expression of both IDO1 and COX-2 is increased in tumor cells and may interface with increased activity of TNF-related apoptosis-inducing ligand (TRAIL) [191]. The AHR is a key mediator in the relationship between kynurenine and COX, including the ability of NFκB to mediate the induction of IDO via COX2. This chain of events contributes to the involvement of IDO in TLR3 activation by viral RNA in the tumor microenvironment [192]. In addition, kynurenine acts via AHR to degrade the adhesion molecule E-cadherin, resulting in enhanced tumor invasion, which is reduced by 1-MT, probably consistent with a role for IDO1 or IDO2. Overall, it was concluded that the combination of increased tumor COX-2 and IDO1 in the microenvironment contributed substantially to the poor prognosis and enhanced metastasis formation in vitro and in vivo.

When the relationship between the kynurenine pathway and COX is under discussion, the question arises of the part played by reactive oxygen species (ROS). Oxidative stress is a common factor between IDO activity, kynurenine metabolism and the activity of NFκB, inflammatory cytokines, TNF, nuclear factor erythroid 2-related factor 2 (Nrf2) and COX-2, with expression of all of these being modulated by the antioxidant indole CMI (3-(4-chlorophenyl)selanyl)-1-methyl-1H-indole (CMI) in mammary-tumor-bearing mice [193]. As an inhibitor of both iNOS and COX-2, these effects of CMI suggest that the modulation of ROS might be a primary candidate for the initiating mechanism. The discovery that kynureninase is regulated by Nrf2 is of great interest, as it reinforces the links between kynurenine production and the redox environment [194]. Frequently expressed in cancer cells, the parallel over-activity of Nrf2 and kynureninase parallels the suppression of host immunity and a reduction in patient survival. 

The increased eicosanoid synthesis in tumors is exemplified by acute myeloid leukemia cells. IFNγ induced both IDO1 and COX-2 expression, but COX-2 inhibition (using nimesulide) prevented induction of IDO1 [195], indicating an essential role of COX-2 in activating the kynurenine pathway. Although inhibition of the PD-1/PD-L1 axis has proved very valuable, some melanomas and models have proved resistant. Tumor growth and progression were reduced by increased effector T cell invasion when COX-2 expression was prevented [196], resulting in a parallel reduction in PD-L1 and IDO1 levels in the tumor microenvironment, which were jointly responsible for the tumor survival.

One of the major COX products responsible for this relationship is prostaglandin-D2 (PGD2) acting on the DP1 receptor and its activation of protein kinase A and CREB (Cyclic AMP Response Element Binding protein) [197]. PGA2 also contributes to this, but PGE2 is inactive [198]. On the other hand, there is evidence that PGE2 can upregulate TDO2, at least in human glioma cell lines, associated with increased expression of its receptor EP4 [199]. Consistent with this, the overexpression of COX-2 described in mammary tumor cells is associated with increased IDO1 and kynurenine levels [200] in co-cultured fibroblasts. Surprisingly, arachidonic acid itself interferes with the IFNγ and STAT3 induction of IDO1, and compounds are being developed to reproduce this phenomenon by mimicking the inhibitory effect of arachidonate on IDO1 expression [201]. 

The interface between COX-2 and IDO is important for the immune system activity of autonomic function. Epinephrine (adrenaline) has little immunosuppressant activity alone, but it potentiates COX-2 and IDO1 expression by TNF, with macrophage-induced T cell suppression [202]. The COX-2 inhibitor celecoxib prevented the induction of IDO1, consistent with previous data [203,204,205]. This relationship seems to occur in parallel with the inhibition of IDO1 expression by STAT1 and Interferon Regulatory Factor 1 (IRF-1) ligation [206]. 

The combined action of IFNγ and TNF was found to induce a suppression of type-1 immunity to a degree far greater than could be seen using either agent alone [207]. There was also a synergistic induction of COX-2, but inhibition of this enzyme precluded the effects of the two cytokines, resulting in enhanced immunity in patients with ovarian cancer. The clear implication that COX-2 inhibition could potentiate the efficacy of anti-cancer treatments would be of some importance.

Given the prominent role of IFNγ in the immune system, interest has been extended to the study of Stimulators of Interferon-γ (STING) molecules. When mice bearing established Lewis lung carcinomas were treated with synthetic cyclic di-adenyl monophosphate (CDA) to activate STING, a rapid increase in immune regulatory pathways was observed involving PD-1, IDO and COX-2 in the tumor micro-environment [208]. PD-1 blockade enhanced anti-tumor responses to CDA and increased mouse survival but did not eliminate the primary tumor. In contrast, the combination of CDA and celecoxib controlled tumor growth, enhanced survival and induced resistance to tumor re-challenge.

### 4.5. HIF-1α

Hypoxia and its induction of HIF-1α play a significant role in the progression of tumors [209]. Extracting database information on hypoxia-related genes led to the identification of three genes (PPFIA4, SERPINE1 and STC2), expression of which was increased in hypoxic patients. They were associated with worse pathology of colonic cancers, higher levels of checkpoint molecules and reduced tumor infiltration by CD8+ T cells but higher numbers of Tregs [210]. Huang et al. [211] implicated VHL-HIF-1α in regulating the differentiation of Th1 and T follicular helper cells using in vivo CRISPR technology to define potential therapeutic targets. One of these may be TDO, as HIF-1α has been found to inhibit this enzyme [212]. 

### 4.6. Pyruvate Dehydrogenase Lipoamide Kinase Isozyme 1 (PDK1)

Human pyruvate dehydrogenase lipoamide kinase isozyme-1 (PDK1) is a mitochondrial enzyme, and the related enzyme pyruvate dehydrogenase (PDH) is a key contributor to the oxidative decarboxylation of pyruvate, which is inhibited by phosphorylation. The PDK1 pathway has become of particular interest because of its association with FOXO1 and the kynurenine pathway. 

A link between the kynurenines and PDK1 was established in a study of Th2 cells [64], which revealed that 3-HAA interferes with PDK1 phosphorylation and its inhibition of NFκB. The interaction was seen specifically in CD4+Tc and in cells activated by TCR activity, with the resulting death of activated Th2 cells. Thus, 3-HAA inhibits NFκB activity, leading to Th2 cell death. When investigated in adipose tissue in relation to the development of obesity and diabetes, it was found that deleting PDK1 in mice produced insulin resistance and glucose intolerance in adipose cells, typical of diabetes [65]. These effects could be prevented by the adipose-specific deletion of FOXO1. The relationship between PDK1 and FOXO1 was promoted by insulin and resulted in a reduced production of leukotrienes. The overall effect of insulin was to regulate leukotriene production to sustain sensitivity via a PDK1–FOXO1 pathway. The modulation of PDK1 by 3-HAA could represent a novel but highly significant activity of the kynurenine metabolite.

### 4.7. Status of Drug Development

While there have been efforts for around 30 years to generate clinically useful drugs based around the kynurenine pathway, they have often been thwarted by the parallel increase in knowledge. The first compound of interest, 1-methyl-tryptophan, proved to have a more complex pharmacology than originally realized. The L- and D-isomers show weak binding to IDO1, but the L-isomer is much more active in inhibiting IDO2. Indeed, the D-isomer (indoximod) is more active as an inhibitor of the IDO1 pathway, not by inhibiting the enzyme directly, but by interacting with downstream sites such as mTORC1, which regulates protein synthesis and cell growth in response to nutrient availability and redox status [213]. It also skews the pattern of tryptophan metabolism, with the expected reduction in kynurenine generation but an unexpected increase in kynurenic acid levels [214]. Nevertheless, it has been assessed in clinical trials, many of which have been summarized [215,216]. Table 1 lists trials that have reached Phase 2 and remain in progress for different forms of cancer. 

The second compound that has progressed to clinical trials is epacadostat. This has also proved clinically ineffective in a randomized trial combined with the PD1 pathway inhibitor pembrolizumab in patients with melanoma [127]. Overall, of the many trials of IDO inhibitors initiated in the past decade [215,216], most have not progressed beyond Phase 1, or the results have not been published (Table 1). The shift towards selective inhibitors of IDO2 [217,218], dual inhibitors of IDO1 and IDO2 [37] or dual inhibitors of IDO1/2 and TDO [33,35,219] represents a sensible trend toward a definitive assessment of the value of dioxygenase inhibition in cancer. The selective inhibition of IDO2 will be of particular interest, as it promotes expression of IFNγ and increases effector cell invasion of the tumor microenvironment, suppressing growth and cell migration [220].

Paradoxically, one of the earliest attempts to produce useful compounds was targeted on KMO, with the generation of Ro61-8048 [221]. This remains the standard for inhibiting KMO experimentally and, with interest shifting from the dioxygenases, may yet be the forerunner of clinically important compounds. This is especially so for conditions involving cell loss, such as neurodegeneration or organ failure [222], but may equally well be relevant to the control of some cancers arising from an inflammatory background such as pancreatitis [223].

**Table 1:** Examples of clinical trials in progress at Phase 2 with the first two compounds to enter trials.

Compound	Molecular Target	Trial Target and Phase	Trial ID (NCT)	Reference and Comment
Indoximod (1-methyl-D-tryptophan; NLG-8919)	IDO1 activity inhibition; stimulates mTORC to modulate downstream transduction; promotes kynurenate formation	Metastatic breast cancer Phase 2	NCT02913430	NLG-802 is a prodrug for this, which entered Phase 1 in 2020
Indoximod (1-methyl-D-tryptophan)	As above	Glioblastoma Phase 2	NCT04049669	
Indoximod (1-methyl-D-tryptophan)	As above	Metastatic prostate cancer; Phase 2	NCT01560923	[224] Combination with sipuleucel-T yielded radiological and clinical improvement
Indoximod (1-methyl-D-tryptophan)	As above	Metastatic pancreatic cancer; Phase 2	NCT02077881	[225]
Indoximod (1-methyl-D-tryptophan)	As above	Metastatic breast cancer; Phase 2	NCT01792050	[226] No improvement compared with taxane
Epacadostat INCB024360	IDO1 inhibitor. IC50 = 93 nM in direct enzyme assay; IC50 = 12.5 nM in HeLa cell-based assay	Prostate cancer; Phase 1 and 2	NCT 03493945	[227,228]
Epacadostat INCB024360	As above	Gliomas; Phase 2	NCT03532295	
Epacadostat INCB024360	As above	Endometrial cancer; Phase 2	NCT04463771	
Epacadostat INCB024360	As above	Head and neck cancer; Phase 2	NCT03463161	

## 5. Conclusions

While the principle of inhibiting IDO or targeting alternative sites in the kynurenine pathway continues to provide a recognized anti-cancer option, different or additional targets may be needed. One of the problems is the need to affect the kynurenine pathway selectively in specific cell populations, and while this may be feasible using bi-specific targeting antibodies or gene-targeting methods, this could be a major pharmacological challenge. This review has attempted to survey other molecules or pathways that affect the kynurenine pathway or are affected by it. Modulating kynurenine metabolism directly, or indirectly by interfering with an interacting pathway that is altered in tumor cells, could provide a conceptual approach worth developing. Certainly, since an earlier overview of T cell pharmacology [229], the direction of research and the range of potential targets has expanded substantially, but the principle and validity of modulating T cell differentiation in the treatment of cancers has become established [230]. 

## References

[1] Huang Y.S., Ogbechi J., Clanchy F., Williams R.O., Stone T.W. (2020). Kynurenine metabolites in peripheral disorders and neuroinflammation. Front. Immunol..

[2] Guillemin G.J., Meininger V., Brew B.J. (2005). Implications for the kynurenine pathway and quinolinic acid in amyotrophic lateral sclerosis. Neurodegener. Dis..

[3] Andersson J., Tran D.Q., Pesu M., Davidson T.S., Ramsey H., O’Shea J.J., Shevach E.M. (2008). CD4(+)FoxP3(+) regulatory T cells confer infectious tolerance in a TGF-beta-dependent manner. J. Exp. Med..

[4] Belladonna M.L., Volpi C., Bianchi R., Vacca C., Orabona C., Pallotta M.T., Boon L., Gizzi S., Fioretti M.C., Grohmann U. (2008). Cutting edge: Autocrine TGF-beta sustains default tolerogenesis by IDO-competent dendritic cells. J. Immunol..

[5] Belladonna M.L., Orabona C., Grohmann U., Puccetti P. (2009). TGF-β and kynurenines as the key to infectious tolerance. Trends Mol. Med..

[6] Yoshida R., Imanishi J., Oku T., Kishida T., Hayaishi O. (1981). Induction of pulmonary indoleamine 2,3-dioxygenase by interferon. Proc. Natl. Acad. Sci. USA.

[7] Yasui H., Takai K., Yoshida R., Hayaishi O. (1986). Interferon enhances tryptophan metabolism by inducing pulmonary indoleamine 2,3-dioxygenase: Its possible occurrence in cancer patients. Proc. Natl. Acad. Sci. USA.

[8] Stone T.W., Clanchy F.I.L., Huang Y.-S., Chiang N.Y., Darlington L.G., Williams R.O. (2022). An integrated cytokine and kynurenine network as the basis of neuroimmune communication. Front. Neurosci..

[9] Sinclair L.V., Neyens D., Ramsay G., Taylor P.M., Cantrell D.A. (2018). Single cell analysis of kynurenine and System L amino acid transport in T cells. Nat. Commun..

[10] Ogbechi J., Wright H.L., Balin S., Topping L.M., Kristina Z., Huang Y.S., Pantazi E., Swart M., Windell D., Marin E. (2023). LAT1 enables T cell activation under inflammatory conditions: A new therapeutic target for rheumatoid arthritis. J. Autoimmun..

[11] Ogbechi J., Huang Y.-S., Clanchy F.I.L., Pantazi E., Topping L.M., Darlington L.G., Williams R.O., Stone T.W. (2022). Modulation of immune cell function; IDO expression and kynurenine production by the quorum sensor 2-heptyl-3-hydroxy-4-quinolone (PQS). Front. Immunol..

[12] Stone T.W., Perkins M.N. (1981). Quinolinic acid: A potent endogenous excitant at amino acid receptors in CNS. Eur. J. Pharmacol..

[13] Stone T.W. (1993). The neuropharmacology of quinolinic and kynurenic acids. Pharmacol. Rev..

[14] Perkins M.N., Stone T.W. (1982). An iontophoretic investigation of the action of convulsant kynurenines and their interaction with the endogenous excitant quinolinic acid. Brain Res..

[15] Perkins M.N., Stone T.W. (1985). Actions of kynurenic acid and quinolinic acid in the rat hippocampus in vivo. Exp. Neurol..

[16] Yue Y., Huang W., Liang J., Guo J., Ji J., Yao Y., Zheng M.Z., Cai Z.J., Lu L.R., Wang J.L. (2015). IL4I1 Is a Novel Regulator of M2 macrophage polarization that can inhibit T cell activation via L-tryptophan and arginine depletion and IL-10 production. PLoS ONE.

[17] Sadik A., Patterson L.F.S., Ozturk S., Mohapatra S.R., Panitz V., Secker P.F. (2020). IL4I1 Is a Metabolic Immune Checkpoint that Activates the AHR and Promotes Tumor Progression. Cell.

[18] Hezaveh K., Shinde R.S., Klotgen A., Halaby M.J., Lamorte S., Ciudad M.T., Quevedo R., Neufeld L., Liu Z.Q., Jin R. (2022). Tryptophan-derived microbial metabolites activate the aryl hydrocarbon receptor in tumor-associated macrophages to suppress anti-tumor immunity. Immunity.

[19] Boulland M.L., Marquet J., Molinier-Frenkel V., Moller P., Guiter C., Lasoudris F., Copie-Berman C., Baia M., Gaulard P., Leroy K. (2007). Human IL4I1 is a secreted L-phenylalanine oxidase expressed by mature dendritic cells that inhibits T-lymphocyte proliferation. Blood.

[20] Mason J.M., Naidu M.D., Barcia M., Porti D., Chavan S.S., Chu C.C. (2004). IL-4-induced gene-1 is a leukocyte L-amino acid oxidase with an unusual acidic pH preference and lysosomal localization. J. Immunol..

[21] Marquet J., Lasoudris F., Cousin C., Puiffe M.L., Martin-Garcia N., Baud V., Chereau F., Farcet J.P., Molinier-Frenkel V., Castellano F. (2020). Dichotomy between factors inducing the immunosuppressive enzyme IL-4-induced gene 1 (IL4I1) in B lymphocytes and mononuclear phagocytes. Eur. J. Immunol..

[22] Carbonnelle-Puscian A., Copie-Bergman C., Baia M., Martin-Garcia N., Allory Y., Haioun C., Cremades A., Abd-Alsamad I., Farcet J.P., Gaulard P. (2009). The novel immunosuppressive enzyme IL4I1 is expressed by neoplastic cells of several B-cell lymphomas and by tumor-associated macrophages. Leukemia.

[23] Lasoudris F., Cousin C., Prevost-Blondel A., Martin-Garcia N., Abd-Alsamad I., Ortonne N., Farcet J.P., Castellano F., Molinier-Frenkel V. (2011). IL4i1: An inhibitor of the CD8(+) antitumor T-cell response in vivo. Eur. J. Immunol..

[24] Fallarino I., Grohmann U., Vacca C., Bianchi R., Orabona C., Spreca A., Fioretti M.C., Puccett P. (2002). T cell apoptosis by tryptophan catabolism. Cell Death Differ..

[25] Fallarino F., Grohmann U., You S., McGrath B.C., Cavener D.R., Vacca C., Orabona C., Bianchi R., Belladonna M.L., Volpi C. (2006). The combined effects of tryptophan starvation and tryptophan catabolites down-regulate T cell receptor zeta-chain and induce a regulatory phenotype in naive T cells. J. Immunol..

[26] Terness P., Bauer T.M., Rose L., Dufter C., Watzlik A., Simon H., Opelz G. (2002). Inhibition of allogeneic T cell proliferation by indoleamine 2,3-dioxygenase-expressing dendritic cells: Mediation of suppression by tryptophan metabolites. J. Exp. Med..

[27] Liu H., Huang L., Bradley J., Liu K., Bardhan K., Ron D., Mellor A.L., Munn D.H., McGaha T.L. (2014). GCN2-dependent metabolic stress is essential for endotoxemic cytokine induction and pathology. Mol. Cell. Biol..

[28] Zhou L., Lopes J.E., Mark M.W., Chong M.M.W., Ivanov I.I., Min R., Victora D., Shen Y., Du J., Rubtsov Y.P. (2008). TGF-β-induced Foxp3 inhibits TH17 cell differentiation by antagonizing RORct function. Nature.

[29] Cribbs A.P., Kennedy A., Penn H., Read J.E., Amjadi P., Green P., Syed K., Manka S.W., Brennan F.M., Gregory B. (2014). Treg Cell Function in Rheumatoid Arthritis Is Compromised by CTLA-4 Promoter Methylation Resulting in a Failure to Activate the Indoleamine 2,3-Dioxygenase Pathway. Arthritis Rheumatol..

[30] Prendergast G.C., Metz R., Muller A.J., Merlo L.M.F., Mandik-Nayak L. (2014). IDO2 in immunomodulation and autoimmune disease. Front. Immunol..

[31] Merlo L.M.F., DuHadaway J.B., Montgomery J.D., Peng W.D., Murray P.J., Prendergast G.C., Caton A.J., Muller A.J., Mandik-Nayak L. (2020). Differential Roles of IDO1 and IDO2 in T and B Cell Inflammatory Immune Responses. Front. Immunol..

[32] Merlo L.M.F., Peng W., DuHadaway J.B., Montgomery J.D., Prendergast G.C., Muller A.J., Mandik-Nayak L. (2022). The Immunomodulatory Enzyme IDO2 Mediates Autoimmune Arthritis through a Nonenzymatic Mechanism. J. Immunol..

[33] Pan S., Zhou Y., Wang Q.S., Wang Y.L., Tian C.Y., Wang T.Q., Huang L.Y., Nan J.S., Li L.L., Yang S.Y. (2020). Discovery and structure-activity relationship studies of 1-aryl-1H-naphtho[2,3-d][1,2,3]triazole-4,9-dione derivatives as potent dual inhibitors of indoleamine 2,3-dioxygenase 1 (IDO1) and tryptophan 2,3-dioxygenase (TDO). Eur. J. Med. Chem..

[34] Pan L., Zheng Q., Chen Y., Yang R., Yang Y., Li Z.J., Meng X.B. (2018). Design, synthesis and biological evaluation of novel naphthoquinone derivatives as IDO1 inhibitors. Eur. J. Med. Chem..

[35] Cui G.N., Lai F.F., Wang X.Y., Chen X.G., Xu B.L. (2020). Design, synthesis and biological evaluation of indole-2-carboxylic acid derivatives as IDO1/TDO dual inhibitors. Eur. J. Med. Chem..

[36] Serafini M., Torre E., Aprile S., Del Grosso E., Gesu A., Griglio A., Colombo G., Travelli C., Paiella S., Adamo A. (2020). Discovery of highly potent benzimidazole derivatives as indoleamine 2,3-dioxygenase-1 (ido1) inhibitors: From structure-based virtual screening to in Vivo Pharmacodynamic Activity. J. Med. Chem..

[37] He X., He G., Chu Z.X., Wu H., Wang J., Ge Y.R., Shen H., Zhang S., Shan J.X., Peng K.W. (2021). Discovery of the First Potent IDO1/IDO2 Dual Inhibitors: A Promising Strategy for Cancer Immunotherapy. J. Med. Chem..

[38] Zhang W., Zhang Q., Yang N.F., Shi Q., Su H.F., Lin T.S., He Z.L., Wang W.X., Guo H.Q., Shen P.P. (2022). Crosstalk between IL-15R alpha(+) tumor-associated macrophages and breast cancer cells reduces CD8(+) T cell recruitment. Cancer Commun..

[39] Delpoux A., Michelini R.H., Verma S., Lai C.Y., Omilusik K.D., Utzschneider D.T., Redwood A.J., Goldrath A.W., Benedict C.A., Hedricj S.M. (2018). Continuous activity of Foxo1 is required to prevent anergy and maintain the memory state of CD8(+) T cells. J. Exp. Med..

[40] Jancewicz I., Szarkowska J., Konopinski R., Stachowiak M., Swiatek M., Blachnio K., Kubala S., Oksinska P., Konopinski R., Stachowiak M. (2021). PD-L1 Overexpression, SWI/SNF Complex Deregulation, and Profound Transcriptomic Changes Characterize Cancer-Dependent Exhaustion of Persistently Activated CD4(+) T Cells. Cancers.

[41] Campos J.S., Henrickson S.E. (2022). Defining and targeting patterns of T cell dysfunction in inborn errors of immunity. Front. Immunol..

[42] Ford B.R., Vignali P.D.A., Rittenhouse N.L., Scharping N.E., Peralta R., Lontos K., Frisch A.T., Delgoffe G.M., Poholek A.C. (2022). Tumor microenvironmental signals reshape chromatin landscapes to limit the functional potential of exhausted T cells. Sci. Immunol..

[43] Wirthgen E., Hoeflich A., Rebl A., Guenther J. (2018). Kynurenic Acid: The Janus-Faced Role of an immunomodulatory tryptophan metabolite and its link to pathological conditions. Front. Immunol..

[44] Walczak K., Langner E., Makuch-Kocka A., Szelest M., Szalast K., Marciniak S., Plech T. (2020). Effect of tryptophan-derived AHR ligands; kynurenine; kynurenic acid and FICZ; on proliferation; cell cycle regulation and cell death of melanoma cells-in vitro studies. Int. J. Mol. Sci..

[45] Walczak K., Wnorowski A., Turski W.A., Plech T. (2020). Kynurenic acid and cancer: Facts and controversies. Cell. Mol. Life Sci..

[46] Tiszlavicz Z., Németh B., Fülöp F., Vécsei L., Tápai K., Ocsovszky I., Mandi Y. (2011). Different inhibitory effects of kynurenic acid and a novel kynurenic acid analogue on tumour necrosis factor-α (TNF-α) production by mononuclear cells; HMGB1 production by monocytes and HNP1-3 secretion by neutrophils. Arch. Pharmacol..

[47] Wang J., Simonavicius N., Wu X., Swaminath G., Reagan J., Tian H., Ling L. (2006). Kynurenic acid as a ligand for orphan G protein-coupled receptor GPR35. J. Biol. Chem..

[48] Resta F., Masi A., Sili M., Laurino A., Moroni F., Mannaioni G. (2016). Kynurenic acid and zaprinast induce analgesia by modulating HCN channels through GPR35 activation. Neuropharmacology.

[49] Wyant G.A., Yu W., Doulamis I.P., Nomoto R.S., Saeed M.Y., Duignan T., McCully J.D., Kaelin W.G. (2022). Mitochondrial remodeling and ischemic protection by G protein-coupled receptor 35 agonists. Science.

[50] Kvaratskhelia E., Maisuradze E., Dabrundashvili N.G., Natsvlishvili N., Zhuravliova E., Mikeladze D.G. (2009). N-Methyl-D-Aspartate and sigma-ligands change the production of interleukins 8 and 10 in lymphocytes through modulation of the NMDA glutamate receptor. Neuro-Immunomodulation.

[51] Lombardi G., Dianzani C., Miglio G., Canonico P.L., Fantozzi R. (2001). Characterization of ionotropic glutamate receptors in human lymphocytes. Br. J. Pharmacol..

[52] Bhandage A.K., Jin Z., Hellgren C., Korol S.V., Nowak K., Williamsson L., Sundstrom-Poromaa I., Birnir B. (2017). AMPA, NMDA and kainate glutamate receptor subunits are expressed in human peripheral blood mononuclear cells (PBMCs) where the expression of GluK4 is altered by pregnancy and GluN2D by depression in pregnant women. J. Neuroimmunol..

[53] Mashkina A.P., Cizkova D., Vanicky I., Boldyrev A.A. (2010). NMDA receptors are expressed in lymphocytes activated both in vitro and in vivo. Cell. Mol. Neurobiol..

[54] Boldyrev A.A., Kazey V.I., Leinsoo T.A., Mashkina A.P., Tyulina O.V., Johnson P., Tuneva J.O., Chittur S. (2004). Rodent lymphocytes express functionally active glutamate receptors. Biochem. Biophys. Res. Commun..

[55] Boldyrev A.A., Bryushkova E.A., Vladychenskaya E.A. (2012). NMDA Receptors in Immune Competent Cells. Biochemistry.

[56] Nohara L.L., Stanwood S.R., Omilusik K.D., Jefferies W.A. (2015). Tweeters; woofers and horns: The complex orchestration of calcium currents in T lymphocytes. Front. Immunol..

[57] Turski W.A., Gramsbergen J.B., Traitler H., Schwarcz R. (1989). Rat brain slices produce and liberate kynurenic acid upon exposure to L-kynurenine. J. Neurochem..

[58] Heredi J., Cseh E.K., Berko A.M., Veres G., Zadori D., Toldi J., Kis Z., Vecsei L., Ono E., Gellert L. (2019). Investigating KYNA production and kynurenergic manipulation on acute mouse brain slice preparations. Brain Res. Bull..

[59] Correale J. (2021). Immunosuppressive Amino-Acid Catabolizing Enzymes in Multiple Sclerosis. Front. Immunol..

[60] Gargaro M., Vacca C., Massari S., Scalisi G., Manni G., Mondanelli G., Mazza E.M.C., Bicciato S., Pallotta M.T., Orabona C. (2019). Engagement of nuclear coactivator 7 by 3-hydroxyanthranilic acid enhances activation of aryl hydrocarbon receptor in immunoregulatory dendritic cells. Front. Immunol..

[61] Oh G.S., Pae H.O., Choi B.M., Chae S.C., Lee H.S., Ryu D.G., Chung H.T. (2004). 3-Hydroxyanthranilic acid, one of metabolites of tryptophan via indoleamine 2,3-dioxygenase pathway, suppresses inducible nitric oxide synthase expression by enhancing heme oxygenase-1 expression. Biochem. Biophys. Res. Commun..

[62] Gan G.F., Shi Z.P., Liu D., Zhang S.Y., Zhu H., Wang Y.G., Mi J. (2021). 3-hydroxyanthranilic acid increases the sensitivity of hepatocellular carcinoma to sorafenib by decreasing tumor cell stemness. Cell Death Discov..

[63] Gan G., Shi Z., Shangguan C., Zhang J., Yuan Y., Chen L., Liu W., Meng S., Xiong W., Mi J. (2021). The kynurenine derivative 3-HAA sensitizes hepatocellular carcinoma to sorafenib by upregulating phosphatases. Theranostics.

[64] Hayashi T., Mo J.H., Gong X., Rossetto C., Jang A., Beck L., Elliott G.I., Kufareva I., Abagyan R., Broide D.H. (2007). 3-Hydroxyanthranilic acid inhibits PDK1 activation and suppresses experimental asthma by inducing T cell apoptosis. Proc. Natl. Acad. Sci. USA.

[65] Hosooka T., Hosokawa Y., Matsugi K., Shinohara M., Senga Y., Tamori Y., Aoki C., Matsui S., Sasaki T., Kitamura T. (2020). The PDK1-FoxO1 signaling in adipocytes controls systemic insulin sensitivity through the 5-lipoxygenase- leukotriene B-4 axis. Proc. Natl. Acad. Sci. USA.

[66] Zuo H., Tell G.S., Vollset S.E., Ueland P.M., Nygard O., Midttun O., Meyer K., Ulvik A., Eussen S.J.P.M. (2014). Interferon-gamma-Induced Inflammatory Markers and the Risk of Cancer: The Hordaland Health Study. Cancer.

[67] Dugue P.A., Hodge A.M., Ulvik A., Ueland P.M., Midttun O., Rinaldi S., Macinnis R.J., Li S.X., Meyer K., Navionis A.S. (2022). Association of Markers of Inflammation, the Kynurenine Pathway and B Vitamins with Age and Mortality, and a Signature of Inflammaging. J. Gerontol. A Biol. Sci. Med. Sci..

[68] Clanchy F.I.L., Huang I.-S., Ogbechi J., Darlington L.G., Williams R.O., Stone T.W. (2022). Induction of IDO1 and Kynurenine by Serine Proteases Subtilisin, Prostate Specific Antigen, CD26 and HtrA: A New Form of Immunosuppression?. Front. Immunol..

[69] Forrest C.M., McNair K., Vincenten M.C., Darlington L.G., Stone T.W. (2016). Selective depletion of tumour suppressors Deleted in Colorectal Cancer (DCC) and neogenin by environmental and endogenous serine proteases: Linking diet and cancer. BMC Cancer.

[70] McNair K., Forrest C.M., Vincenten M., Darlington L.G., Stone T.W. (2019). Serine protease modulation of dependence receptors and EMT protein expression. Cancer Biol. Ther..

[71] Stone T.W. (2020). Dependence and Guidance Receptors—DCC and neogenin—In partial EMT and the actions of serine proteases. Front. Oncol..

[72] Feng X., Shen P., Wang Y., Li Z.Y., Bian J. (2019). Synthesis and in vivo antitumor evaluation of an orally active potent phosphonamidate derivative targeting IDO1/IDO2/TDO. Biochem. Pharmacol..

[73] Fatokun A.A., Hunt N.H., Ball H.J. (2013). Indoleamine 2,3-dioxygenase 2 (IDO2) and the kynurenine pathway: Characteristics and potential roles in health and disease. Amino Acids.

[74] Huang Y.S., Tseng W.Y., Clanchy F., Topping L.M., Ogbechi J., McNamee K., Perocheau D., Chiang N.Y., Ericsson P., Sundstedt A. (2021). Pharmacological modulation of T cell immunity results in long-term remission of autoimmune arthritis. Proc. Natl. Acad. Sci. USA.

[75] Mondanelli G., Coletti A., Greco F.A., Pallotta M.T., Orabona C., Iacono A., Belladonna M.L., Albini E., Panfili E., Fallarino F. (2020). Positive allosteric modulation of indoleamine 2,3-dioxygenase 1 restrains neuroinflammation. Proc. Natl. Acad. Sci. USA.

[76] Peng Y.P., Zhang J.J., Liang W.B., Tu M., Lu Z.P., Wei J.S., Jiang K.R., Gao W.T., Wu J.L., Xu Z.K. (2014). Elevation of MMP-9 and IDO induced by pancreatic cancer cells mediates natural killer cell dysfunction. BMC Cancer.

[77] Mitra D., Horick N.K., Brackett D.G., Mouw K.W., Hornick J.L., Ferrone S., Hong T.S., Mamon H., Clark J.W., Parikh A.R. (2019). High IDO1 expression is associated with poor outcome in patients with anal cancer treated with definitive chemoradiotherapy. Oncologist.

[78] Jin H.J., Zhang Y.R., You H.Y., Tao X.M., Wang C., Jin G.Z., Wang N., Ruan H.Y., Gu D.S., Huo X.S. (2015). Prognostic significance of kynurenine 3-monooxygenase and effects on proliferation, migration, and invasion of human hepatocellular carcinoma. Sci. Rep..

[79] Forrest C.M., Khalil O.S., Pisar M., Darlington L.G., Stone T.W. (2013). Prenatal inhibition of the tryptophan-kynurenine pathway alters synaptic plasticity and protein expression in the rat hippocampus. Brain Res..

[80] Zhou L.L., Mu L., Jiang W.Y., Yang Q. (2022). QPRT Acts as an Independent Prognostic Factor in Invasive Breast Cancer. J. Oncol..

[81] Ala M. (2021). The footprint of kynurenine pathway in every cancer: A new target for chemotherapy. Eur. J. Pharmacol..

[82] Opitz C.A., Litzenburger U.M., Sahm F., Ott M., Tritschler I., Trump S., Schumacher T., Jestaedt L., Schrenk D., Weller M. (2011). An endogenous tumour-promoting ligand of the human aryl hydrocarbon receptor. Nature.

[83] Bessede A., Gargaro M., Pallotta M.T., Matino D., Servillo G., Brunacci C., Bicciato S., Mazza E.M.C., Macchiarulo A., Vacca C. (2014). Aryl hydrocarbon receptor control of a disease tolerance defence pathway. Nature.

[84] Bankoti J., Rase B., Simones T., Shepherd D.M. (2010). Functional and phenotypic effects of AhR activation in inflammatory dendritic cells. Toxicol. Appl. Pharmacol..

[85] Litzenburger U.M., Opitz C.A., Sahm F., Rauschenbach K.J., Trump S., Winter M., Ott M., Ochs K., Lutz C., Liu X.D. (2014). Constitutive IDO expression in human cancer is sustained by an autocrine signaling loop involving IL-6; STAT3 and the AHR. Oncotarget.

[86] Li Q., Harden J.L., Anderson C.D., Egilmez N.K. (2016). Tolerogenic phenotype of IFN-gamma-induced IDO+ dendritic cells is maintained via an autocrine IDO-kynurenine/AhR-IDO loop. J. Immunol..

[87] Wang Z., Monti S., Sherr D.H. (2017). The diverse and important contributions of the AHR to cancer and cancer immunity. Curr. Opin. Toxicol..

[88] Novikov O., Wang Z., Stanford E.A., Parks A.J., Ramirez-Cardenas A., Landesman E., Laklouk I., Sarita-Reyes C., Gusenleitner D., Li A. (2016). An Aryl Hydrocarbon Receptor-Mediated Amplification Loop That Enforces Cell Migration in ER-/PR-/Her2(-) Human Breast Cancer Cells. Mol. Pharmacol..

[89] Gargaro M., Manni G., Scalisi G., Puccetti P., Fallarino F. (2021). Tryptophan metabolites at the crossroad of immune-cell interaction via the aryl hydrocarbon receptor: Implications for tumor immunotherapy. Int. J. Mol. Sci..

[90] Pallotta M.T., Fallarino F., Matino D., Macchiarula A., Orabona C. (2014). AhR-mediated, non-genomic modulation of IDO1 function. Front. Immunol..

[91] Cheong J.E., Sun L.J. (2018). Targeting the IDO1/TDO2-KYN-AhR Pathway for Cancer Immunotherapy—Challenges and Opportunities. Trends Pharmacol. Sci..

[92] Opitz C.A., Patterson L.F.S., Mohapatra S.R., Dewi D.L., Sadik A., Platten M., Trump S. (2020). The therapeutic potential of targeting tryptophan catabolism in cancer. Br. J. Cancer.

[93] Ehrlich A.K., Pennington J.M., Bisson W.H., Kolluri S.K., Kerkvliet N.I. (2018). TCDD, FICZ, and Other High Affinity AhR Ligands Dose-Dependently Determine the Fate of CD41 T Cell Differentiation. Toxicol. Sci..

[94] Yu L.J., Croze E., Yamaguchi K.D., Tran T., Reder A.T., Litvak V., Volkert M.R. (2015). Induction of a Unique Isoform of the NCOA7 Oxidation Resistance Gene by Interferon beta-1b. J. Interferon Cytokine Res..

[95] Xiong L., Dean J.W., Fu Z., Oliff K.N., Bostick J.W., Ye J., Chen Z., Muhlbauer M., Zhou L. (2020). Ahr-Foxp3-ROR gamma-t axis controls gut homing of CD4(+) T cells by regulating GPR15. Sci. Immunol..

[96] Ye J., Qiu J., Bostick J.W., Ueda A., Schjerven H., Li S., Jobin C., Chen Z.E., Zhou L. (2017). The Aryl Hydrocarbon Receptor Preferentially Marks and Promotes Gut Regulatory T Cells. Cell Rep..

[97] Ohnmacht C., Park J.H., Cording S., Wing J.B., Atarashi K., Obata Y., Gaboriau-Routhiau V., Marques R., Dulauroy S., Fedoseeva M. (2015). The microbiota regulates type 2 immunity through RORgamma(+) T cells. Science.

[98] Swaminathan G., Nguyen L.P., Namkoong H., Pan J., Haileselassie Y., Patel A., Ji A.R., Mikhail D.M., Dinh T., Singh H. (2021). The aryl hydrocarbon receptor regulates expression of mucosal trafficking receptor GPR15. Mucosal Immunol..

[99] Kim S.V., Xiang W.V., Kwak C., Yang Y., Lin X.W., Ota M., Sarpel U., Rifkin D.B., Xu R., Littman D.R. (2013). GPR15-mediated homing controls immune homeostasis in the large intestine mucosa. Science.

[100] Nguyen L.P., Pan J., Dinh T.T., Hadeiba H., O’Hara E., Ebtikar A., Hertweck A., Gokmen M.R., Lord G.M., Jenner R.G. (2015). Role and species-specific expression of colon T cell homing receptor GPR15 in colitis. Nat. Immunol..

[101] Rudra D., de Roos P., Chaudhry A., Niec R.E., Arvey A., Samstein R.M., Leslie C., Shaffer S.A., Goodlett D.R., Rudensky A.Y. (2012). Transcription factor Foxp3 and its protein partners form a complex regulatory network. Nat. Immunol..

[102] Lu L., Barbi J., Pan F. (2017). The regulation of immune tolerance by FOXP3. Nat. Rev. Immunol..

[103] Nagai Y., Lam L., Greene M.I., Zhang H.T. (2019). FOXP3 and Its Cofactors as Targets of Immunotherapies. Engineering.

[104] Samstein R.M., Arvey A., Josefowicz S.Z., Peng X., Reynolds A., Sandstrom R., Neph S., Sabo P., Kim J.M., Liao W. (2012). Foxp3 exploits a pre- existent enhancer landscape for regulatory T cell lineage specification. Cell.

[105] Pavlick K.P., Ostanin D.V., Furr K.L., Laroux F.S., Brown C.M., Gray L., Kevil C.G., Grisham M.B. (2006). Role of T-cell-associated lymphocyte function-associated antigen-1 in the pathogenesis of experimental colitis. Intern. Immunol..

[106] Wolchok J.D. (2015). PD-1 blockers. Cell.

[107] Day C.L., Kaufmann D.E., Kiepiela P., Brown J.A., Moodley E.S., Reddy S., Mackey E.W., Miller E.W., Leslie A.J., DePierres C. (2006). PD-1 expression on HIV-specific T cells is associated with T-cell exhaustion and disease progression. Nature.

[108] Barber D.L., Wherry E.J., Masopust D., Zhu B., Allison J.P., Sharpe A.H., Freeman G.J., Ahmed R. (2006). Restoring function in exhausted CD8 T cells during chronic viral infection. Nature.

[109] Cao W., Lu J., Li L., Qiu C., Qin X., Wang T., Li S., Zhang J., Xu J. (2022). Activation of the Aryl Hydrocarbon Receptor Ameliorates Acute Rejection of Rat Liver Transplantation by Regulating Treg Proliferation and PD-1 Expression. Transplantation.

[110] Baumeister S.H., Freeman G.J., Dranoff G., Sharpe A.H. (2016). Coinhibitory pathways in immunotherapy for cancer. Ann. Rev. Immunol..

[111] Sakuishi K., Apetoh L., Sullivan J.M., Blazar B.R., Kuchroo V.K., Anderson A.C. (2010). Targeting Tim-3 and PD-1 pathways to reverse T cell exhaustion and restore anti-tumor immunity. J. Exp. Med..

[112] Ye Q., Wang C., Xian J., Zhang M., Cao Y., Cao Y. (2018). Expression of programmed cell death protein 1 (PD-1) and indoleamine 2,3-dioxygenase (IDO) in the tumor microenvironment and in tumor draining lymph nodes of breast cancer. Hum. Pathol..

[113] Chen Y.C., He X.L., Qi L., Shi W., Yuan L.W., Huang M.Y., Xu Y.L., Chen X., Gu L., Zhang L.L. (2022). Myricetin inhibits interferon-gamma-induced PD-L1 and IDO1 expression in lung cancer cells. Biochem. Pharmacol..

[114] Anzai H., Yoshimoto S., Okamura K., Hiraki A., Hashimoto S. (2022). IDO1-mediated Trp-kynurenine-AhR signal activation induces stemness and tumor dormancy in oral squamous cell carcinomas. Oral Sci. Int..

[115] Carbotti G., Barisione G., Airoldi I., Mezzanzanica D., Bagnoli M., Ferrero S., Petretto A., Fabbi M., Ferrini S. (2015). IL-27 induces the expression of IDO and PD-L1 in human cancer cells. Oncotarget.

[116] Donizy P., Wu C.L., Kopczynski J., Pieniazek M., Biecek P., Rys J., Hoang M.P. (2021). Prognostic Role of Tumoral PD-L1 and IDO1 Expression, and Intratumoral CD8+ and FoxP3+Lymphocyte Infiltrates in 132 Primary Cutaneous Merkel Cell Carcinomas. Int. J. Mol. Sci..

[117] Xiang Z., Zhou Z.J., Song S.Z., Li J., Ji J., Yan R.L., Wang J.X., Cai W., Hu W.J., Zang L. (2021). Dexamethasone suppresses immune evasion by inducing GR/STAT3 mediated downregulation of PD-L1 and IDO1 pathways. Oncogene.

[118] Kotecki N., Vuagnat P., O’Neil B.H., Jalal S., Rottey S., Prenen H., Benhadj K.A., Xia M., Szpurka A.M., Saha A. (2021). A Phase I Study of an IDO-1 Inhibitor (LY3381916) as Monotherapy and in Combination with an Anti-PD-L1 Antibody (LY3300054) in Patients With Advanced Cancer. J. Immunother..

[119] Jung M.Y., Aibaidula A., Brown D.A., Himes B.T., Garcia L.M.C., Parney I.F. (2022). Superinduction of immunosuppressive glioblastoma extracellular vesicles by IFN-gamma through PD-L1 and IDO1. Neuro-Oncol. Adv..

[120] Kjeldsen J.W., Lorentzen C.L., Martinenaite E., Ellebaek E., Donia M., Holmstroem R.B., Klausen T.W., Madsen C.O., Ahmed S.M., Weis-Banke S.E. (2021). A phase 1/2 trial of an immune-modulatory vaccine against IDO/PD-L1 in combination with nivolumab in metastatic melanoma. Nat. Med..

[121] Abdulla M., Alexsson A., Sundstrom C., Ladenvall C., Mansouri L., Lindskog C., Berglund M., Cavelier L., Enblad G., Hollander P. (2021). PD-L1 and IDO1 are potential targets for treatment in patients with primary diffuse large B-cell lymphoma of the CNS. Acta Oncol..

[122] Liu Y., Liang X., Dong W.Q., Fang Y., Lv J.D., Zhang T.Z., Fiskesund R., Xie J., Liu J.Y., Yin X.N. (2018). Tumor-repopulating cells induce PD-1 expression in CD8+ T cells by transferring kynurenine and AhR activation. Cancer Cell.

[123] Amobi-McCloud A., Muthuswamy R., Battaglia S., Yu H., Liu T., Wang J., Putluri V., Singh P.K., Qian F., Huang R. (2021). IDO1 Expression in Ovarian Cancer Induces PD-1 in T cells via Aryl Hydrocarbon Receptor Activation. Front. Immunol..

[124] Long G.V., Dummer R., Humid O., Gajewski T.F., Caglevic C., Dalle S., Arance A., Carlino M.S., Grob J.J., Kim T.M. (2019). Epacadostat plus pembrolizumab versus placebo plus pembrolizumab in patients with unresectable or metastatic melanoma (ECHO-301/KEYNOTE-252): A phase 3, randomised, double-blind study. Lancet Oncol..

[125] Iwasaki T., Kohashi K., Toda Y., Ishihara S., Yamada Y., Oda Y. (2021). Association of PD-L1 and IDO1 expression with JAK-STAT pathway activation in soft-tissue leiomyosarcoma. J. Cancer Res. Clin. Oncol..

[126] Sabharwal S.S., Rosen D.B., Grein J., Tedesco D., Joyce-Shaikh B., Ueda R., Semana M., Bauer M., Bang K., Stevenson C. (2018). GITR Agonism Enhances Cellular Metabolism to Support CD8(+) T-cell Proliferation and Effector Cytokine Production in a Mouse Tumor Model. Cancer Immunol. Res..

[127] Ishihara S., Yamada Y., Iwasaki T., Yoshimoto M., Toda Y., Kohashi K., Yamamoto H., Matsumoto Y., Nakashima Y., Oda Y. (2021). PD-L1 and IDO-1 expression in undifferentiated pleomorphic sarcoma: The associations with tumor infiltrating lymphocytes; dMMR and HLA class I. Oncol. Rep..

[128] Takada K., Toyokawa G., Kinoshita F., Jogo T., Kohashi K., Wakasu S., Ono Y., Tanaka K., Oba T., Osoegawa A. (2020). Expression of PD-L1, PD-L2, and IDO1 on tumor cells and density of CD8-positive tumor-infiltrating lymphocytes in early-stage lung adenocarcinoma according to histological subtype. J. Cancer Res. Clin. Oncol..

[129] Toda Y., Kohashi K., Yamada Y., Yoshimoto M., Ishihara S., Ito Y., Iwasaki T., Yamamoto H., Matsumoto Y., Nakashima Y. (2020). PD-L1 and IDO1 expression and tumor-infiltrating lymphocytes in osteosarcoma patients: Comparative study of primary and metastatic lesions. J. Cancer Res. Clin. Oncol..

[130] Swainson L.A., Ahn H., Pajanirassa P., Khetarpal V., Deleage C., Estes J.D., Hunt P.W., Munoz-Sanjuan I.G., McCune J. (2019). Kynurenine 3-Monooxygenase Inhibition during Acute Simian Immunodeficiency Virus Infection Lowers PD-1 Expression and Improves Post-Combination Antiretroviral Therapy CD4(+) T cell Counts and Body Weight. J. Immunol..

[131] Miyazaki T., Chung S.Y., Sakai H., Ohata H., Obata Y., Shiokawa D., Mizoguchi Y., Kubo T., Ishikawa H., Taniguchi H. (2022). Stemness and immune evasion conferred by the TDO2-AHR pathway are associated with liver metastasis of colon cancer. Cancer Sci..

[132] Ludovini V., Bianconi F., Siggillino A., Vannucci J., Baglivo S., Berti V., Tofanetti F.R., Reda M.S., Bellezza G., Mandarano M. (2021). High PD-L1/IDO-2 and PD-L2/IDO-1 co-expression levels are associated with worse overall survival in resected non-small cell lung cancer patients. Genes.

[133] Hacking S., Chavarria H., Jin C., Perry A., Nasim M. (2020). Landscape of Immune Checkpoint Inhibition in Carcinosarcoma (MMMT): Analysis of IDO-1; PD-L1 and PD-1. Pathol. Res. Pract..

[134] Tao B.B., Shi J.H., Shuai S., Zhou H.Y., Zhang H.X., Li B., Wang X.Q., Li G.H., He H., Zhong J. (2021). CYB561D2 up-regulation activates STAT3 to induce immunosuppression and aggression in gliomas. J. Transl. Med..

[135] Han Y., Liu D., Li L. (2020). PD-1/PD-L1 pathway: Current researches in cancer. Am. J. Cancer Res..

[136] Bally A.P.R., Tang Y., Lee J.T., Barwick B.G., Martinez R., Evavold B.D., Boss J.M. (2017). Conserved Region C Functions To Regulate PD-1 Expression and Subsequent CD8 T Cell Memory. J. Immunol..

[137] Liu Q., Chen Y., Auger-Messier M., Molkentin J.D. (2012). Interaction Between NFκB and NFAT Coordinates Cardiac Hypertrophy and Pathological Remodeling. Circ. Res..

[138] Liu Y., Shi J.Z., Jiang R., Liu S.F., He Y.Y., van der Vorst E.P.C., Weber C., Doring Y., Yan Y. (2022). Regulatory T cell-related gene indicators in pulmonary hypertension. Front. Pharmacol..

[139] Hsu F.T., Chen T.C., Chuang H.Y., Chang Y.F., Hwang J.J. (2015). Enhancement of adoptive T cell transfer with single low dose pretreatment of doxorubicin or paclitaxel in mice. Oncotarget.

[140] Kaiser H., Parker E., Hamrick M.W. (2020). Kynurenine Signaling through the Aryl Hydrocarbon Receptor: Implications for Aging and Healthspan. Exp. Gerontol..

[141] Welz B., Bikker R., Junemann J., Christmann M., Neumann K., Weber M., Hoffmeister L., Preuss K., Pich A., Huber R. (2019). Proteome and Phosphoproteome Analysis in TNF Long Term-Exposed Primary Human Monocytes. Int. J. Mol. Sci..

[142] D’Amato N.C., Rogers T.J., Gordon M.A., Greene L.I., Cochrane D.R., Spoelstra N.S., Nemkov T.G., D’Alessandro A., Hansen K.C., Richer J.K. (2015). A TDO2-AhR Signaling Axis Facilitates Anoikis Resistance and Metastasis in Triple-Negative Breast Cancer. Cancer Res..

[143] Lee C.H., Bae J.H., Choe E.J., Park J.M., Park S.S., Cho H.J., Song B.J., Baek M.C. (2022). Macitentan improves antitumor immune responses by inhibiting the secretion of tumor-derived extracellular vesicle PD-L1. Theranostics.

[144] Moshofsky K.B., Cho H.J., Wu G.M., Romine K.A., Newman M.T., Kosaka Y., McWeeney S.K., Lind E.F. (2019). Acute myeloid leukemia-induced T-cell suppression can be reversed by inhibition of the MAPK pathway. Blood Adv..

[145] Guan L., Wu B., Li T., Beer L.A., Sharma G., Li M., Lee C.N., Liu S.J., Yang C.S., Huang L.L. (2022). HRS phosphorylation drives immunosuppressive exosome secretion and restricts CD8(+) T-cell infiltration into tumors. Nat. Commun..

[146] Liu J., Kang R., Kroemer G., Tang D.L. (2022). Targeting HSP90 sensitizes pancreas carcinoma to PD-1 blockade. Oncoimmunology.

[147] Yang A.O., Li M.Y., Zhang Z.H., Wang J.Y., Xing Y., Ri M., Jin C.H., Xu G.H., Piao L.X., Jin H.L. (2021). Erianin regulates programmed cell death ligand 1 expression and enhances cytotoxic T lymphocyte activity. J. Ethnopharmacol..

[148] Ando S., Araki K. (2022). CD8 T-cell heterogeneity during T-cell exhaustion and PD-1-targeted immunotherapy. Int. Immunol..

[149] Bae J., Hideshima T., Zhang G.L., Zhou J., Keskin D.B., Munshi N.C., Anderson K.C. (2018). Identification and characterization of HLA-A24-specific XBP1; CD138 (Syndecan-1) and CS1 (SLAMF7) peptides inducing antigens-specific memory cytotoxic T lymphocytes targeting multiple myeloma. Leukemia.

[150] Bae J., Hideshima T., Tai Y.-T., Song Y., Richardson P., Raje N., Munshi N.C., Anderson K.C. (2018). Histone deacetylase (HDAC) inhibitor ACY241 enhances anti-tumor activities of antigen-specific central memory cytotoxic T lymphocytes against multiple myeloma and solid tumors. Leukemia.

[151] Zammarchi F., Havenith K., Bertelli F., Vijayakrishnan B., Chivers S., van Berkel P.H. (2020). CD25-targeted antibody-drug conjugate depletes regulatory T cells and eliminates established syngeneic tumors via antitumor immunity. J. Immunother. Cancer..

[152] Kim C.H. (2007). Molecular targets of FoxP3(+) regulatory T cells. Mini-Rev. Med. Chem..

[153] Bacchetta R., Barzaghi F., Roncarolo M.G. (2018). From IPEX syndrome to FOXP3 mutation: A lesson on immune dysregulation. Ann. N.Y. Acad. Sci..

[154] Kwon H.K., Chen H.M., Mathis D., Benoist C. (2018). FoxP3 scanning mutagenesis reveals functional variegation and mild mutations with atypical autoimmune phenotypes. Proc. Natl. Acad. Sci. USA.

[155] Kwon H.K., Chen H.M., Mathis D., Benoist C. (2017). Different molecular complexes that mediate transcriptional induction and repression by Foxp3. Nat. Immunol..

[156] van Loosdregt J., Coffer P.J. (2014). Post-translational modification networks regulating FOXP3 function. Trends Immunol..

[157] Nakahira K., Morita A., Kim N.S., Yanagihara I. (2013). Phosphorylation of FOXP3 by LCK downregulates MMP9 expression and represses cell invasion. PLoS ONE.

[158] Deng G., Nagai Y., Xiao Y., Li Z., Dai S., Ohtani T., Banham A., Li B., Wu S.L., Hancock W. (2015). Pim-2 kinase influences regulatory T cell function and stability by mediating Foxp3 protein N-terminal phosphorylation. J. Biol. Chem..

[159] Li Z., Lin F., Zhuo C., Deng G., Chen Z., Yin S., Gao Z.M., Piccioni M., Tsun A., Cai S.J. (2014). Pim1 kinase phosphorylates the human transcription factor FOXP3 at serine 422 to negatively regulate its activity under inflammation. J. Biol. Chem..

[160] Luszczak S., Simpson B.S., Stopka-Farooqui U., Sathyadevan V.K., Echeverria L.M.C., Kumar C., Costa H., Haider A., Freeman A., Jameson C. (2020). Co-targeting PIM and PI3K/mTOR using multikinase inhibitor AUM302 and a combination of AZD-1208 and BEZ235 in prostate cancer. Sci. Rep..

[161] Tanaka S., Pfleger C., Lai J.F., Roan F., Sun S.C., Ziegler S.F. (2018). KAP1 regulates regulatory T cell function and proliferation in both FOXP3-dependent and -independent manners. Cell Rep..

[162] Lozano T., Gorraiz M., Lasarte-Cia A., Ruiz M., Rabal O., Oyarzabal J., Hervas-Stubbs S., Llopiz D., Sarobe P., Prieto J. (2017). Blockage of FOXP3 transcription factor dimerization and FOXP3/AML1 interaction inhibits T regulatory cell activity: Sequence optimization of a peptide inhibitor. Oncotarget.

[163] Liu X., Ji B., Sun M., Wu W., Huang L., Sun A., Zong Y., Xia S., Shi L., Qian H. (2015). Cell-penetrable mouse Forkhead box protein 3 alleviates experimental arthritis in mice by up-regulating regulatory T cells. Clin. Exp. Immunol..

[164] Wang Z., Qi Y., Feng Y., Xu H., Wan J., Zhang L., Zhang J., Hou X., Feng G., Shang E. (2022). The N6-methyl-adenosine writer WTAP contributes to the induction of immune tolerance post kidney transplantation by targeting regulatory T cells. Lab. Investig..

[165] Zeng R., Peng B., Peng E.M. (2022). Downregulated copper homeostasis-related gene FOXO1 as a novel indicator for the prognosis and immune response of breast cancer. J. Immunol. Res..

[166] Wang D., Yang L., Yu W., Wu Q., Lian J., Li F., Liu S., Li A., He Z., Liu J. (2019). Colorectal cancer cell-derived CCL20 recruits regulatory T cells to promote chemoresistance via FOXO1/CEBPB/NF-kappa B signaling. J. Immunother. Cancer.

[167] Luu T.T., Sondergaard J.N., Pena-Perez L., Kharazi S., Krstic A., Meinke S., Schmied L., Frengen N., Heshmati Y., Kierczak M. (2022). FOXO1 and FOXO3 Cooperatively Regulate Innate Lymphoid Cell Development. Front. Immunol..

[168] Kesarwani P., Kant S., Zhao Y., Prabhu A., Buelow K.L., Miller C.R., Chinnaiyan P. (2023). Quinolinate promotes macrophage-induced immune tolerance in glioblastoma through the NMDA/PPARγ signaling axis. Nat. Commun..

[169] Zhang T., Zhang Z., Li F., Ping Y., Qin G., Zhang C., Zhang Y. (2018). miR-143 Regulates Memory T cell Differentiation by Reprogramming T cell Metabolism. J. Immunol..

[170] Huang Q., Xia J., Wang L., Wang X., Ma X., Deng Q., Lu Y., Kumar M., Zhou Z.Y., Li L. (2018). miR-153 suppresses IDO1 expression and enhances CAR T cell immunotherapy. J. Haematol. Oncol..

[171] Lou Q., Liu R., Yang X., Li W., Huang L., Wei L., Tan H., Xiang N., Chan K., Chen J. (2019). miR-448 targets IDO1 and regulates CD8(+) T cell response in human colon cancer. J. Immunother. Cancer.

[172] van der Windt G.J.W., Pearce E.L. (2012). Metabolic switching and fuel choice during T-cell differentiation and memory development. Immunol. Rev..

[173] van der Windt G.J.W., Everts B., Chang C.H., Curtis J.D., Freitas T.C., Amiel E., Pearce E.J., Pearce E.L. (2012). Mitochondrial Respiratory Capacity Is a Critical Regulator of CD8(+) T Cell Memory Development. Immunity.

[174] Gao J., Liu Y., Wei J., Jiang L., Mao J., Chang C.H., Wu D. (2021). Targeting T cell metabolism for immunotherapy. J. Leukoc. Biol..

[175] Tan S.Y., Kelkar Y., Hadjipanayis A., Shipstone A., Wynn T.A., Hall J.P. (2020). Metformin and 2-Deoxyglucose Collaboratively Suppress Human CD4(+) T Cell Effector Functions and Activation-Induced Metabolic Reprogramming. J. Immunol..

[176] Maciver N.J., Jacobs S.R., Wieman H.L., Wofford J.A., Coloff J.L., Rathmell J.C. (2008). Glucose metabolism in lymphocytes is a regulated process with significant effects on immune cell function and survival. J. Leukoc. Biol..

[177] Li W.H., Xu M., Li Y., Huang Z.W., Zhou J., Zhao Q.Y., Le K.H., Dong F., Wan C., Yi P.F. (2020). Comprehensive analysis of the association between tumor glycolysis and immune/inflammation function in breast cancer. J. Transl. Med..

[178] Dimeloe S., Burgener A.V., Grahlert J., Hess C. (2017). T-cell metabolism governing activation, proliferation and differentiation; a modular view. Immunology.

[179] Chang C.H., Curtis J.D., Maggi L.B., Faubert B., Villarino A.V., O’Sullivan D., Huang S.C.C., can der Windt G.J.W., Blagih J., Qiu J. (2013). Post-transcriptional Control of T Cell Effector Function by Aerobic Glycolysis. Cell.

[180] Kalyanaraman B., Cheng G., Hardy M. (2022). Therapeutic Targeting of Tumor Cells and Tumor Immune Microenvironment Vulnerabilities. Front. Oncol..

[181] Balmer M.L., Hess C. (2017). Starving for survival-how catabolic metabolism fuels immune function. Curr. Opin. Immunol..

[182] Frauwirth K.A., Thompson C.B. (2004). Regulation of T lymphocyte metabolism. J. Immunol..

[183] Fox C.J., Hammerman P.S., Thompson C.B. (2005). Fuel feeds function: Energy metabolism and the T-cell response. Nat. Rev. Immunol..

[184] Gubser P.M., Bantug G.R., Razik L., Fischer M., Dimeloe S., Hoenger G., Durovic B., Jauch A., Hess C. (2013). Rapid effector function of memory CD8+ T cells requires an immediate-early glycolytic switch. Nat. Immunol..

[185] Garige M., Ghosh S., Norris A., Li G.Y., Poncet S., Chou C.K., Wu W.W., Shen R.F., Sourbier C. (2022). PD-L1 Mediates IFN gamma-Regulation of Glucose but Not of Tryptophan Metabolism in Clear Cell Renal Cell Carcinoma. Front. Oncol..

[186] Toriyama K., Kuwahara M., Kondoh H., Mikawa T., Takemori N., Konishi A., Yorozuya T., Yamada T., Soga T., Shiraishi A. (2020). T cell-specific deletion of *Pgam1* reveals a critical role for glycolysis in T cell responses. Commun. Biol..

[187] Li G., Liu L., Yin Z., Ye Z., Shen N. (2021). Glutamine metabolism is essential for the production of IL-17A in gamma-delta T cells and skin inflammation. Tissue Cell.

[188] Yu Q., Tu H., Yin X., Peng C., Dou C., Yang W., Wu W., Guan X., Li J., Yan H. (2022). Targeting glutamine metabolism ameliorates autoimmune hepatitis via inhibiting T cell activation and differentiation. Front. Immunol..

[189] Wu J., Li G., Li L., Li D., Dong Z., Jiang P. (2021). Asparagine enhances LCK signalling to potentiate CD8(+) T-cell activation and anti-tumour responses. Nat. Cell Biol..

[190] Hope H., Brownlie R.J., Fife M., Steele L., Lorger M., Salmond R.J. (2021). Coordination of asparagine uptake and asparagine synthetase expression modulates CD8(+) T cell activation. JCI Insight.

[191] Wesch D., Kabelitz D., Oberg H.H. (2020). Tumor resistance mechanisms and their consequences on gamma delta T cell activation. Immunol. Rev..

[192] Theodoraki M.N., Yerneni S., Sarkar S.N., Orr B., Muthuswamy R., Voyten J., Modugno F., Jiang W.J., Grimm M., Bsse P.H. (2018). Helicase-Driven Activation of NF kappa B-COX2 Pathway Mediates the Immunosuppressive Component of dsRNA-Driven Inflammation in the Human Tumor Microenvironment. Cancer Res..

[193] Casaril A.M., Domingues M., Bampi S.R., Lourenco D.D., Smaniotto T.A., Segatto N., Vieira B., Seixas F.K., Collares T., Lemardao E.J. (2020). The antioxidant and immunomodulatory compound 3-[(4-chlorophenyl)selanyl]-1-methyl-1H-indole attenuates depression-like behavior and cognitive impairment developed in a mouse model of breast tumor. Brain Behav. Immun..

[194] Leon-Letelier R.A., Sater A.A.H., Chen Y.H., Park S., Wu R.R., Irajizad E., Dennison J.B., Katayama H., Vykoukal J.V., Hanash S. (2023). Kynureninase Upregulation Is a Prominent Feature of NFR2-Activated Cancers and Is Associated with Tumor Immunosuppression and Poor Prognosis. Cancers.

[195] Iachininoto M.G., Nuzzolo E.R., Bonanno G., Mariotti A., Procoli A., Locatelli F., DeCristofaro R., Rutella S. (2013). Cyclooxygenase-2 (COX-2) Inhibition Constrains Indoleamine 2,3-Dioxygenase 1 (IDO1) Activity in Acute Myeloid Leukaemia Cells. Molecules.

[196] Erkes D.A., Field C.O., Capparelli C., Tiago M., Purwin T.J., Chervoneva I., Berger A.C., Hartsough E.J., Villanueva J., Aplin A.E. (2019). The next-generation BET inhibitor, PLX51107, delays melanoma growth in a CD8-mediated manner. Pigment Cell Melanoma Res..

[197] Bassal N.K., Hughes B.P., Costabile M. (2016). Prostaglandin D-2 is a novel repressor of IFN gamma induced indoleamine-2,3-dioxygenase via the DP1 receptor and cAMP pathway *Prostaglandins Leukot*. Essent. Fat. Acids.

[198] Bassal N.K., Hughes B.P., Costabile M. (2012). Arachidonic acid and its COX1/2 metabolites inhibit interferon-gamma mediated induction of indoleamine-2,3 dioxygenase in THP-1 cells and Human monocytes. Prostaglandins Leukot. Essent. Fat. Acids.

[199] Ochs K., Ott M., Rauschenbach K.J., Deumelandt K., Sahm F., Opitz C.A., von Deimling A., Wick W., Platten M. (2016). Tryptophan-2,3-dioxygenase is regulated by prostaglandin E2 in malignant glioma via a positive signaling loop involving prostaglandin E receptor-4. J. Neurochem..

[200] Chen J.Y., Li C.F., Kuo C.C., Tsai K.K., Hou M.F., Hung W.C. (2014). Cancer/stroma interplay via cyclooxygenase-2 and indoleamine 2,3-dioxygenase promotes breast cancer progression. Breast Cancer Res..

[201] Costabile M., Bassal N.K., Gerber J.P., Hughes B.P. (2017). Inhibition of indoleamine 2,3-dioxygenase activity by fatty acids and prostaglandins: A structure function analysis. Prostaglandins Leukot. Essent. Fat. Acids.

[202] Muthuswamy R., Okada N.J., Jenkins F.J., McGuire K., McAuliffe P.F., Zeh H.J., Bartlett D.L., Wallace C., Watkins S., Henning J.D. (2017). Epinephrine promotes COX-2-dependent immune suppression in myeloid cells and cancer tissues. Brain Behav. Immun..

[203] Basu G., Tinder T.L., Bradley J.M., Tu T., Hattrup C.L., Pockaj B.A., Mukherjee P. (2006). Cyclooxygenase-2 inhibitor enhances the efficacy of a breast cancer vaccine: Role of IDO. J. Immunol..

[204] Lee S.Y., Choi H.K., Lee K.J., Jung J.Y., Hur G.Y., Jung K.H., Kim J.H., Shin C., Shim J.J., In K.H. (2009). The Immune Tolerance of Cancer is Mediated by IDO That is Inhibited by COX-2 Inhibitors Through Regulatory T cells. J. Immunother..

[205] Cesario A., Rocca B., Rutella S. (2011). The Interplay between Indoleamine 2,3-Dioxygenase 1 (IDO1) andCyclooxygenase (COX)-2 In Chronic Inflammation and Cancer. Current Med. Chem..

[206] Jeong Y.I., Jung I.D., Lee J.S., Lee C.M., Lee J.D., Park Y.M. (2007). (-)-Epigallocatechin gallate suppresses indoleamine 2,3-dioxygenase expression in murine dendritic cells: Evidences for the COX-2 and STAT1 as potential targets. Biochem. Biophys. Res. Commun..

[207] Wong J.L., Obermajer N., Odunsi K., Edwards R.P., Kalinski P. (2016). Synergistic COX2 Induction by IFN gamma and TNF alpha Self-Limits Type-1 Immunity in the Human Tumor Microenvironment. Cancer Immunol. Res..

[208] Lemos H., Ou R., McCardle C., Lin Y.J., Calver J., Minett J., Chadli A., Huang L., Mellor A.L. (2020). Overcoming resistance to STING agonist therapy to incite durable protective antitumor immunity. J. Immunother. Cancer.

[209] Ramsay G., Cantrell D. (2015). Environmental and metabolic sensors that control T cell biology. Front. Immunol..

[210] Xu Y.X., Cao C., Zhu Z., Wang Y., Tan Y., Xu X. (2022). Novel Hypoxia-Associated Gene Signature Depicts Tumor Immune Microenvironment and Predicts Prognosis of Colon Cancer Patients 2022. Front. Genet..

[211] Huang B.N., Phelan J.D., Preite S., Gomez-Rodriguez J., Johansen K.H., Shibata H., Shaffer A.L., Xu Q., Jeffrey B., Kirby M. (2022). In vivo CRISPR screens reveal a HIF-1 alpha-mTOR-network regulates T follicular helper versus Th1 cells. Nat. Commun..

[212] Mohapatra S.R., Sadik A., Tykocinski L.O., Dietze J., Poschet G., Heiland I., Opitz C.A. (2019). Hypoxia Inducible Factor 1-alpha. Front. Immunol..

[213] Prendergast G.C., Malachowski W.P., DuHadaway J.B., Muller A.J. (2017). Discovery of IDO1 Inhibitors: From Bench to Bedside. Cancer Res..

[214] Wirthgen E., Leonard A.K., Scharf C., Domanska G. (2020). The immunomodulatory 1-methyltryptophan drives tryptophan catabolism toward the kynurenic acid branch. Front. Immunol..

[215] Tang K., Wu Y.H., Song Y., Yu B. (2021). Indoleamine 2,3-dioxygenase 1 (IDO1) inhibitors in clinical trials for caner immunotherapy. J. Haematol. Oncol..

[216] Pires A.S., Sundaram G., Heng B., Krishnamurthy S., Brew B.J., Guillemin G.J. (2022). Recent advances in clinical trials targeting the kynurenine pathway. Pharmacol. Ther..

[217] Röhrig U.F., Majjigapu S.R., Caldelari D., Dilek N., Reichenbach P., Ascencao K., Irving M., Coukos J., Vogel P., Zoete V. (2016). 1,2,3-Triazoles as inhibitors of indoleamine 2,3-dioxygenase 2 (IDO2). Bioorg. Med. Chem. Lett..

[218] He G.C., Wan S., Wu Y.Z., Chu Z.X., Shen H., Zhang S., Chen L.Y., Bao Z.J., Gu S.H., Huang J.Z. (2022). Discovery of the First Selective IDO2 Inhibitor as Novel Immunotherapeutic Avenues for Rheumatoid Arthritis. J. Med. Chem..

[219] Zhang Y., Hu Z.L., Zhang J.F., Ren C.Y., Wang Y.X. (2022). Dual-target inhibitors of indoleamine 2, 3 dioxygenase 1 (Ido1): A promising direction in cancer immunotherapy. Eur. J. Med. Chem..

[220] Yamasuge W., Yamamoto Y., Fujigaki H., Hoshi M., Nakamoto K., Kunisawa K., Mouri A., Nabeshima T., Saito K. (2019). Indoleamine 2,3-dioxygenase 2 depletion suppresses tumor growth in a mouse model of Lewis lung carcinoma. Cancer Sci..

[221] Rover S., Cesura A.M., Huguenin P., Kettler R., Szente A. (1997). Synthesis and biochemical evaluation of N-(4-phenylthiazol-2-yl)benzenesulfonamides as high-affinity inhibitors of kynurenine 3-hydroxylase. J. Med. Chem..

[222] Mole D.J., Webster S.P., Uings I., Zheng X.Z., Binnie M., Wilson K., Hutchinson J.P., Mirguet O., Walker A., Beaufils B. (2016). Kynurenine-3-monooxygenase inhibition prevents multiple organ failure in rodent models of acute pancreatitis. Nat. Med..

[223] Walker A.L., Ancellin N., Beaufils B., Bergeal M., Binnie M., Bouillot A., Clapham D., Denis A., Haslan C.P., Holmes D.S. (2017). Development of a Series of Kynurenine 3-Monooxygenase Inhibitors Leading to a Clinical Candidate for the Treatment of Acute Pancreatitis. J. Med. Chem..

[224] Jha G.G., Gupta S., Tagawa S.T., Koopmeiners J.S., Vivek S., Dudek A.Z., Miller J.S. (2017). A phase II randomized, double-blind study of sipuleucel-T followed by IDO pathway inhibitor, indoximod, or placebo in the treatment of patients with metastatic castration resistant prostate cancer (mCRPC). J. Clin. Oncol..

[225] Bahary N., Wang-Gillam A., Haraldsdottir S., Somer B.G., Lee J.S., O’Rourke A.M., Nayak-Kapoor A., Beatty G.L., Liu M., Delman D. (2018). Phase 2 trial of the IDO pathway inhibitor indoximod plus gemcitabine/nab-paclitaxel for the treatment of patients with metastatic pancreas cancer. J. Clin. Oncol..

[226] Mariotti V., Han H., Ismail-Khan R., Tang S.J., Dillon P., Montero A.J., Poklepovic A., Melin S., Ibrahim N.K., Kennedy E. (2021). Effect of Taxane Chemotherapy With or Without Indoximod in Metastatic Breast Cancer A Randomized Clinical Trial. JAMA Oncol..

[227] Redman J.M., Steinbrg S.M., Gulley J.L. (2018). Quick efficacy seeking trial (QuEST1): A novel combination immunotherapy study designed for rapid clinical signal assessment metastatic castration-resistant prostate cancer. J. Immunother. Cancer.

[228] von Amsberg G., Alsdorf W., Karagiannis P., Coym A., Kaune M., Werner S., Graefen M., Bokemeyer C., Merkens L., Dyshlovoy S.A. (2022). Immunotherapy in Advanced Prostate Cancer—Light at the End of the Tunnel?. Int. J. Mol. Sci..

[229] Smethurst D. (2013). A pharmacologic perspective on newly emerging T-cell manipulation technologies. Br. J. Clin. Pharmacol..

[230] Fattori S., Roux H., Connen E., Robert L., Gorvel L., Le Roy A., Houacine J., Foussat A., Chretien A.S., Olive D. (2022). Therapeutic targeting of tumor-infiltrating regulatory T cells in breast cancer. Cancer Res..

